# Mitochondrial Enzymes Mimetic Ultrasmall Palladium Nanozymes Prevent Senescence and Neurodegeneration Through Metabolic Reprogramming

**DOI:** 10.1002/advs.202523931

**Published:** 2026-04-14

**Authors:** Wenshu Cong, Haiming Jing, Zinan Li, Wenjing Zhang, Nan Zhang, Yingqiu Xie, Shan Gao, Yuanyu Huang, Junyu Ning

**Affiliations:** ^1^ Beijing Center for Disease Prevention and Control Beijing Key Laboratory of Diagnostic and Traceability Technologies for Food Poisoning Beijing China; ^2^ School of Life Science School of Interdisciplinary Science Key Laboratory of Molecular Medicine and Biotherapy Key Laboratory of Medical Molecule Science and Pharmaceutics Engineering Beijing Institute of Technology Beijing China; ^3^ School of Public Health Capital Medical University Beijing China; ^4^ Department of Biology, School of Sciences and Humanities Nazarbayev University Astana Kazakhstan; ^5^ School of Medical Engineering School of Interdisciplinary Science Affiliated Zhuhai People's Hospital Beijing Institute of Technology Zhuhai China

**Keywords:** aging, mitochondria, nanozyme, palladium nanoparticles, reactive oxygen species

## Abstract

Nanomaterials have been widely used to scavenge reactive oxygen species (ROS) and relieve mitochondria oxidative damage. However, developing nanomedicines that not only remove ROS but also accelerate the repair of dysfunctional mitochondria remains challenging. This study identifies polyvinylpyrrolidone (PVP)‐modified palladium nanoparticles (PdP NPs) as mimics of cytochrome *c* oxidase (C*c*O) and superoxide dismutase (SOD), showcasing their potential as multifunctional nanoreactors to activate mitochondria for aging alleviation and neuroprotection. PdP NPs treatment enhances mitochondrial respiratory chain function, scavenges excessive ROS, thus alleviates cellular energy scarcity of aging individuals. Additionally, PdP NPs improve mitochondrial dynamics, promote biogenesis, and induce mitochondrial unfolded protein response (UPR^mt^), strengthening mitochondrial integrity and homeostasis for better therapeutic outcomes. In vivo evaluations reveal significant anti‐aging effects, with the nanozymes notably reducing neurodegeneration and improving neuronal survival. This work highlights PdP NPs as a multifunctional nanotherapeutic platform capable of rewiring mitochondrial metabolism and homeostasis, offering a promising strategy for aging‐related disease management.

## Graphical abstract

Simply constructed polyvinylpyrrolidone (PVP) modified palladium nanoparticles (PdP NPs) as cytochrome *c* oxidase (C*c*O) and superoxide dismutase (SOD) mimic are promising multifunctional nanoreactors to boost mitochondrial respiration chain function and refine homeostasis via regulation of mitochondrial dynamics and proteostasis sensor UPR^mt^, which markedly alleviate aging and neurodegeneration in *Caenorhabditis elegans*.

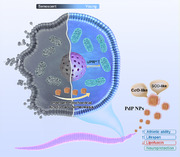

## 1 Introduction

Senescence is associated with progressive physical and functional decline, which poses severe global burden on public health [1, 2, 3]. Exploring efficient treatment strategies based on the aging related pathogenesis is highly critical. Increasing evidence indicate that mitochondrial function extends far beyond energy generation, since a causative link between mitochondrial dysfunction and age‐related diseases [4, 5, 6, 7]. C*c*O, as the terminal metalloenzyme of mitochondrial respiratory chain, can catalyze the four‐electron reduction of oxygen to water and consume more than 90% of the respired oxygen in mammalian cells [8, 9]. It not only represents the oxygen accepting and the rate‐limiting enzyme of the respiratory chain, regulating the supply of variable ATP demands in cell, but also is critically associated with the mitochondrial biogenesis and dynamics [9, 10]. Several studies found that the structural and functional disorders of C*c*O appeared in mitochondrial‐rich tissues such as heart and brain in many aging‐related diseases such as cancer and neurodegeneration [11, 12, 13, 14]. Excessive production of reactive oxygen species (ROS) by the inefficient electron transport during aging can disrupt C*c*O activity and normal electron transport, exacerbate abnormal ROS accumulation, and create a vicious cycle that intensifies mitochondrial dysfunction, leading to metabolic disorder and cellular energy crisis [15, 16, 17]. These facts demonstrated the impairment of C*c*O activity plays a key role in mitochondrial dysfunction in aging related pathogenesis. Superoxide dismutase (SOD) can scavenge ROS by catalyzing the dismutation of superoxide anion free radical $\left(O_{2}^{\cdot -}\right)$ and form the front line of defense [18]. However, natural SOD levels in the body drop as the body ages. Thus, a rational design focused on recovery of C*c*O and SOD activity has shown promising therapeutic importance in markedly improving mitochondrial function and alleviating senescence [19, 20]. Although extracted natural enzymes with high catalytic activity and substrate selectivity are being developed, the intrinsic drawbacks such as high cost of manufacture and storage, complex reconstruction and purification, low recyclability as well as the potential immunogenicity hinder their clinical application [21]. Besides, due to the lack of precise assembly approaches, it is difficult to encapsulate multiple natural enzymes into one vesicle to modulate complex metabolic processes.

In a different line, studies in model organisms have started to integrate effects on cellular senescence with imbalance of mitochondrial homeostasis [22]. Loss of proteostasis along with aging can result in impaired protein quality control and accumulation of misfolded proteins. These facts highlight the importance of homeostasis modulation in directing mitochondrial fate for retarding aging‐related diseases. Maintenance of mitochondrial homeostasis requires both generation of new healthy mitochondrial and elimination of the dysfunctional ones. Multiple mechanisms involved in mitochondrial biogenesis, fusion/fission dynamics and proteostasis have evolved to maintain mitochondrial metabolic homeostasis and promote the health of the organism [22, 23]. Mitochondrial unfolded protein response (UPR^mt^) serves as a master mitochondrial proteostasis pathway that orchestrates an adaptive reprogramming of metabolism for the maintenance of mitochondrial homeostasis and cytoprotection [24, 25]. Similar to other defense systems, compromised UPR^mt^ is identified as a hallmark of several age‐related diseases [24, 25]. However, current strategies to activate UPR^mt^ for alleviating senescence are rather limited.

Nanomaterials with intrinsic enzymatic activity (termed as nanozymes) have attracted extensive interest in the past decade as promising candidates for various disorders due to their unique physicochemical properties, relatively low cost in production, facile synthesis process, high biological stability, ability to mimic natural molecular and potential bioactivity [26, 27, 28, 29, 30]. Currently, most available nanozymes have been reported to exhibit oxidase or peroxidase activity, such as single‐atom Cu–Mn nanozyme for superoxide dismutase (SOD) and catalase (CAT) mimicking activities, PdIr nanocatalysts for superior peroxidase (POD) mimicking activity [30, 31]. Despite of the promising progress, the types of enzymes that can be simulated by nanomaterials are rather limited. For instance, due to the fact that the functional unit of C*c*O is composed of multiple metal cofactor sites and protein subunits, developing synthetic enzymes that can mimic C*c*O is highly challenging [32]. This limitation restricts the application scope and therapeutic effects of nanozymes in complex environments with disease progression. Very recently, some studies found that a few nanomaterials such as CeVO_4_ can serve as C*c*O substitutes and promote mitochondrial electron transport [32]. However, though reasonable and attractive, the effect of nanozymes on restoring dysfunctional mitochondria and metabolic homeostasis to support the repair cells and promote healthy lifespan remains unclear and needs to be further explored.

**FIGURE 1:**
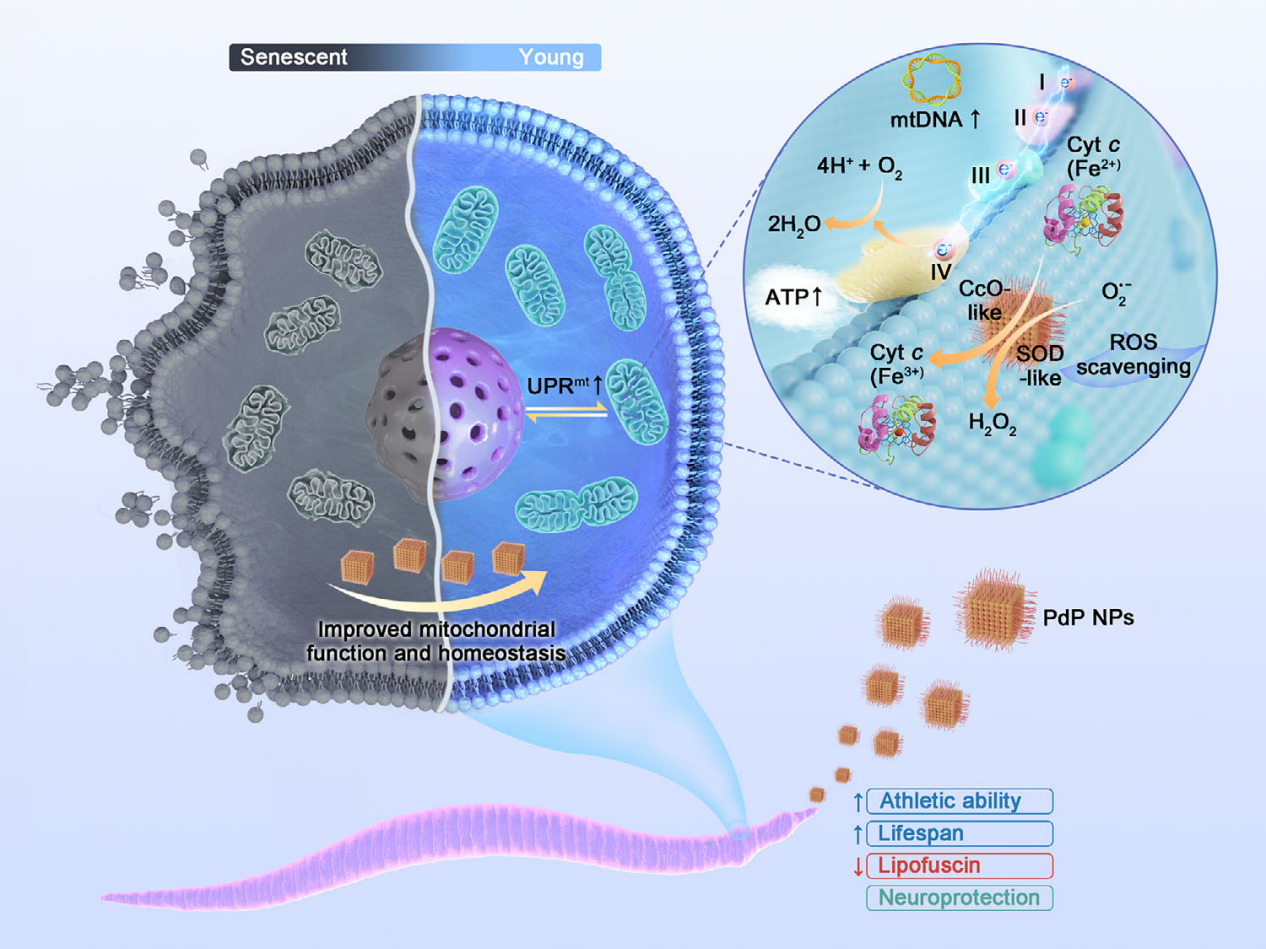
The proposed mechanism by which PdP NPs show nanocatalytic anti‐aging effect on *C. elegans*.

Palladium (Pd) nanomaterials have drawn extensive attention in biomedical applications due to active surface site, simple structure and preparation, superior catalytic potential and potential bioactivity [33, 34, 35, 36]. Here in this paper, we reported the discovery that polyvinylpyrrolidone (PVP) modified ultrasmall palladium nanoparticles (abbreviated as PdP NPs) as C*c*O and SOD mimics, with the intrinsic therapeutic potency of alleviating mitochondrial dysfunction and homeostasis disorder against aging and aging‐related neurodegenerative disease (Figure 1). *Caenorhabditis elegans* (*C. elegans*), which has small size, body transparency, short life cycle, and genetic manipulability, were adopted as models in the vivo study [37]. We proved that PdP NPs not only functionally show high efficiency in boosting energy production but also decrease oxidative stress. Meanwhile, it could effectively refine mitochondrial dynamics and biogenesis as well as activate mitochondrial unfolded protein response (UPR^mt^) pathway, thus maintaining mitochondrial integrity and homeostasis along with aging. More importantly, our in vivo experiments showed the PdP NPs treatment could not only significantly extend lifespan but also simultaneously retard aging‐related behavioral and functional decline, proving its anti‐aging therapeutic efficacy. Furthermore, the nanozymes treatment was capable of realizing alleviation of neurodegenerative disorder and neuronal survival rate increase in *C. elegans*. Our study extends the biomedical applications of PdP NPs as mitochondrial stimulator in the fields of anti‐aging and neuroprotection, as well as provides a new horizon for enzyme‐catalyzed mitochondrial therapy.

## 2 Results and Discussion

### 2.1 Characterization of PdP NPs and Assessment of Multiple Enzyme Activities

**FIGURE 2:**
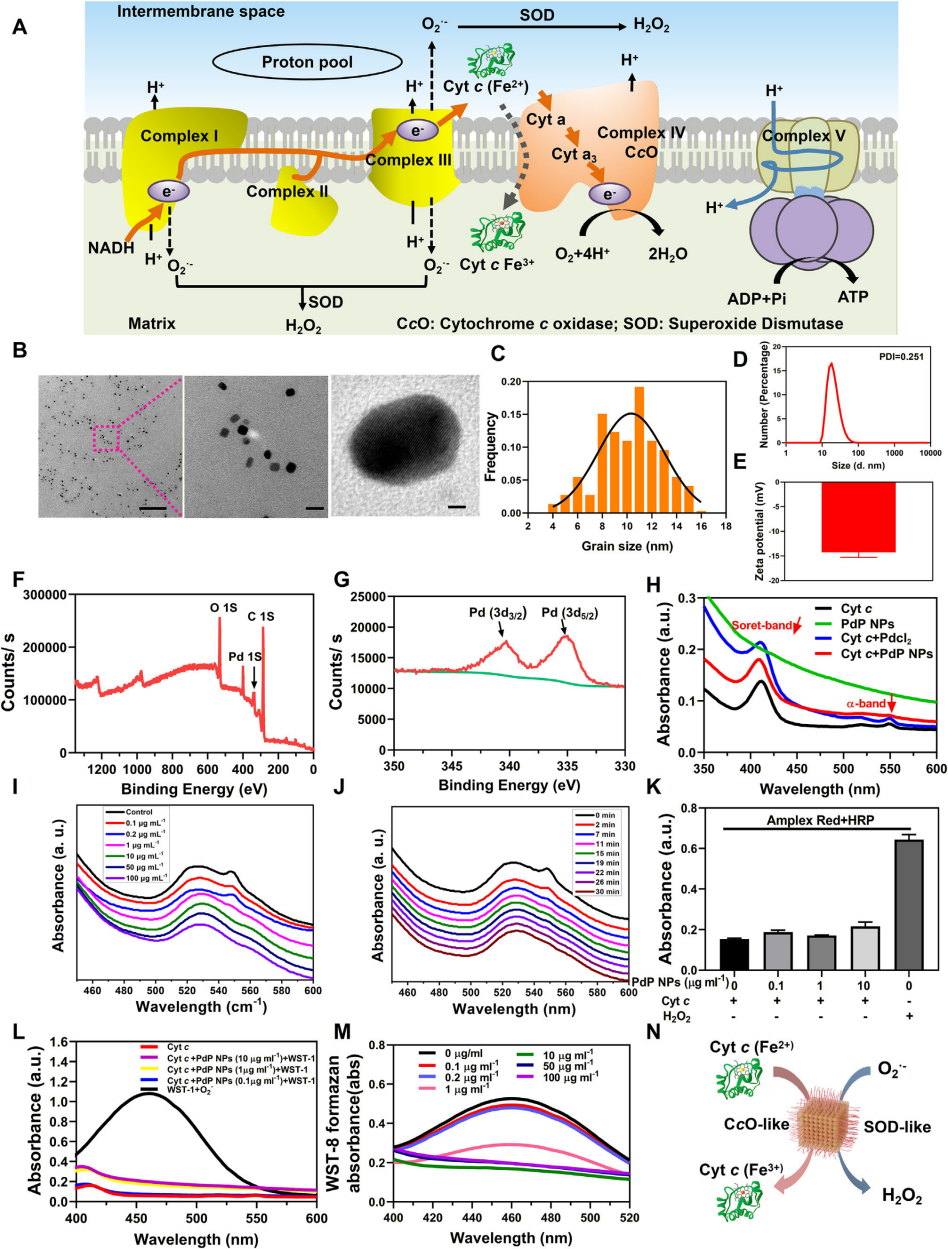
Characterization of enzyme‐mimicking activities of PdP NPs. (A) Schematic diagram of the respiratory electron transport chain of mitochondrial, indicating the role of cytochrome *c* oxidase (C*c*O) and superoxide dismutase (SOD). (B) TEM and HRTEM images of PdP NPs. Scale bars: 200 nm (left), 20 nm (middle) and 2 nm (right). (C) Diameter distribution of PdP NPs measured by TEM. (D,E) Hydrodynamic size and zeta potential of PdP NPs measured by dynamic light scattering (DLS). (F) XPS survey spectrum and (G) High resolution XPS spectrum of the Pd3d region for PdP NPs. (H) The UV–vis absorption spectra of cytochrome *c* after its reaction with PdP NPs or Pdcl_2_ for 30 min. (I) The UV–vis absorption spectra of cytochrome *c* after its reaction with PdP NPs at various concentrations (0.1–100 µg mL^−1^) for 30 min. (J) UV–vis absorption spectra after cytochrome *c* reacted with PdP NPs (1 µg mL^−1^) at different times (0−30 min). (K) Determination of H_2_O_2_ production using Amplex red (*n* = 3). (L) Determination of superoxide production caused by the C*c*O‐like activity of PdP NPs using WST‐1. (M) SOD‐like activity of PdP NPs was tested by WST‐8 kit assay. (N) Schematic representation of PdP NPs that functionally mimic C*c*O and SOD.

To develop multifunctional enzymes that mimic C*c*O and SOD which play vital roles in mitochondrial respiratory chain (Figure 2A), the PdP NPs were constructed by a simple one‐step and mild method described elsewhere using Na_2_PdCl_4_ powders and PVP as the source material. PVP acted both as a reducing agent and a dispersion stabilizer during PdP NPs synthesis. As characterized in transmission electron microscopy (TEM) images, the obtained PdP NPs displayed monodisperse cubelike shape with a side length of 10.1 ± 2.5 nm (Figure 2B,C). The size distributions and zeta potential of PdP NPs dispersed in water were also characterized using dynamic light scattering (DLS), which indicated a uniform size of approximately 30 nm and −14.2 mV (Figure 2D,E). And no obvious change was found in three tested days when PdP NPs was dispersed in aqueous solution, indicating good dispersity and stability of the prepared nanomaterials (Figure S1). In addition, energy‐dispersive X‐ray spectroscopy (EDX) and X‐ray photoelectron spectroscopy (XPS) were employed to characterize the elemental composition of PdP NPs and verify their successful synthesis. As shown in the Figure 2F; Figure S2, abundant signals of Pd and Carbon (C) existed in the survey spectrum. There is a pair of stronger peaks appeared at 334.9 eV (Pd 3d5/2) and 340.3 eV (Pd 3d3/2), which are ascribed to metallic Pd (0) (Figure 2G). Then we detected the C*c*O enzyme‐like activity of PdP NPs. Normally, the ferrous cytochrome *c* (Cyt *c*) is characterized by a Soret band at 414 nm and the a‐band at 550 nm [32]. Oxidation of ferrous Cyt *c* into ferric Cyt *c* by PdP NPs was evidenced by decrease of adsorption peak at 550 nm as well as blue‐shift of the Soret band at 409 nm in a dose‐ and time‐dependent manner (Figure 2H–J). The oxidation of ferrous Cyt *c* could also be confirmed colorimetrically since the color of Cyt *c* alters from pink to brick‐red. Crucially, most of Cyt *c* is oxidized within initial 7 mins, indicating a remarkably high C*c*O activity of PdP NPs (Figure 2J). Insufficient catalysis leads to generation of Partially Reduced Oxygen Species (PROS). To verify whether PdP NPs could induce the possible formation of such species, Amplex Red assay was adopted to exclude the release of H_2_O_2_ by 2e‐reduction. While the reaction of Amplex Red with H_2_O_2_ showed the upregulated fluorescence signal, no fluorescence enhancement was tested obviously in the PdP NPs‐mediated reaction (Figure 2K). Moreover, the superoxide $\left(O_{2}^{\cdot -}\right)$ generation due to 1e‐reduction of dioxygen was ruled out based on an assay using WST‐1 dye, which reacts with $O_{2}^{\cdot -}$ to generate formazan (Figure 2L). Above findings proved that PdP NPs could mediate a complete 4e‐reduction of dioxygen to water without releasing PROS (Figure S3).

In addition to the C*c*O activity, SOD‐like activity of PdP NPs for clearing hydroxyl radical was also detected. The experimental results of this study showed that formazan production decreases with increasing concentrations of PdP NPs (Figure 2M; Figure S4), signifying the SOD‐mimetic antioxidant properties. To sum up, the multiple enzyme‐like activities of PdP NPs were well characterized (Figure 2N), which were expected to improve mitochondrial function and protect mitochondria from the troublesome oxidative stress microenvironment.

### 2.2 PdP NPs Treatment Boost Mitochondrial Function of Aging *C. elegans*

**FIGURE 3:**
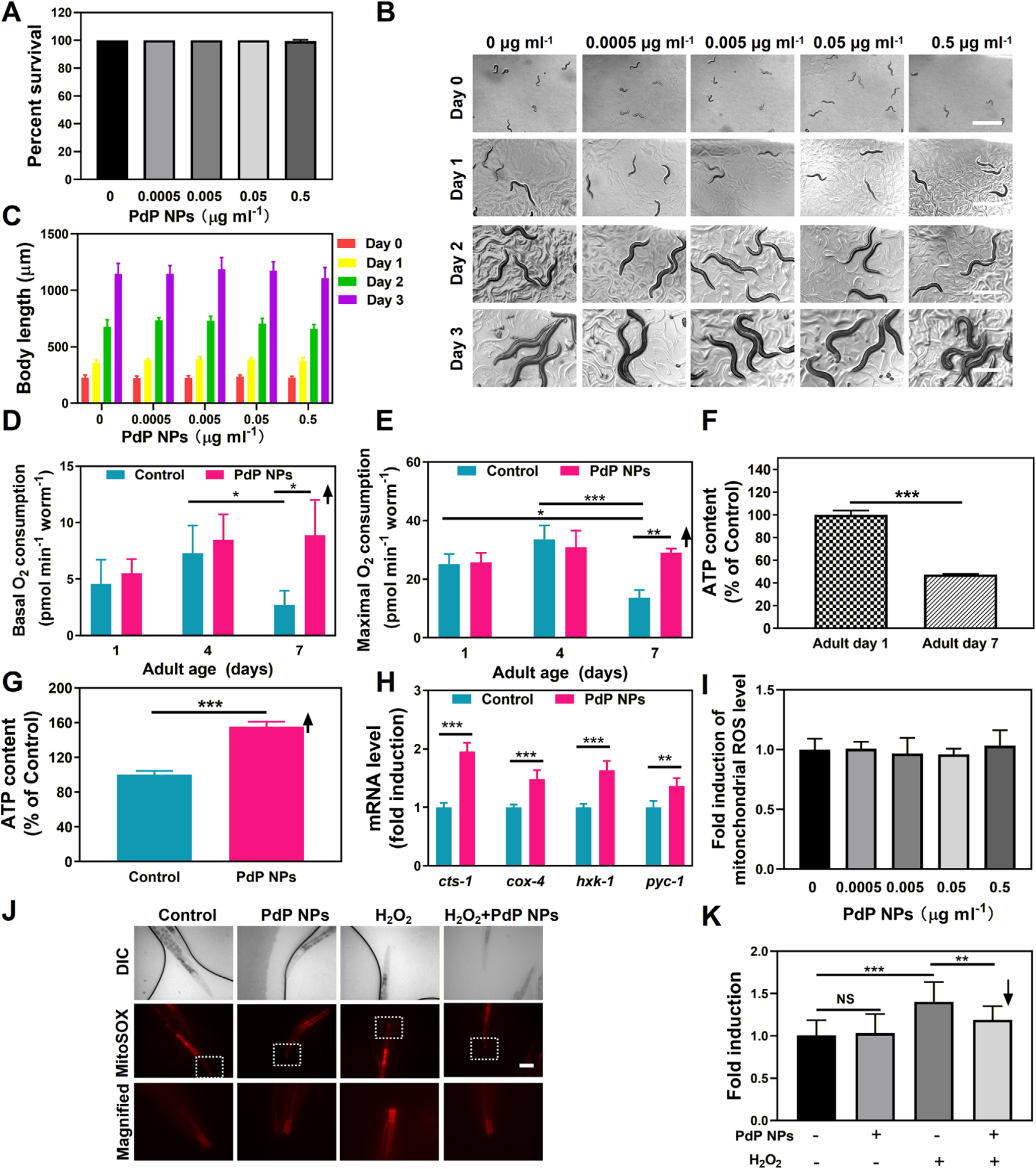
PdP NPs treatment enhance energy metabolism in *C. elegans*. (A) Effects of PdP NPs on worms’ survival rate (*n* = 30‐50; *P*_0.0005_ > 0.9999, *P*_0.005_ > 0.9999, *P*_0.05_ > 0.9999, *P*_0.5_ = 0.5393); (B,C) Effects of PdP NPs on the body growth at different development stage. Representative pics of worms and quantitative comparison of body length were conducted (*n* = 16‐32). *C. elegans* were treated with PdP NPs for three days since L1 stage. (D) Basal oxygen consumption of worms on adult day 1 (AD1), adult day 4 (AD4), and adult day 7 (AD7). *n* = 3; *P* = 0.9415 (AD1 PdP NPs versus AD1 control)_,_ *P* = 0.8499 (AD4 PdP NPs versus AD4 control), *P* = 0.0122 (AD7 PdP NPs versus AD7 control), *P* = 0.0491 (AD7 control versus AD4 control). (E) Maximum oxygen consumption of worms at different development age (*n* = 3). *C. elegans* were treated with PdP NPs for three days since L1 stage. *P* = 0.9968 (AD1 PdP NPs versus AD1 control)_,_ *P* = 0.7436 (AD4 PdP NPs versus AD4 control)_,_ *P* = 0.0019 (AD7 PdP NPs versus AD7 control), *P* = 0.0062 (AD7 control versus AD1 control), *P* < 0.0001 (AD7 control versus AD4 control). (F) Comparison of ATP content in *C. elegans* on days 1 and 7 of adulthood (*n* = 3, *P* < 0.0001). (G) Comparison of ATP levels between PdP NPs treated and mock treated *C. elegans* on day 7 of adulthood (*n* = 3, *P* = 0.0002). (H) The mRNA level of key metabolic genes *cts‐1* (TCA cycle), *cox‐4* (ETC chain enzyme complex IV), *hxk‐1* (glycolysis) and *pyc‐1* (gluconeogenesis) in *C. elegans* after PdP NPs or mock treatment (*n* = 3; *P_cts‐1_* < 0.0001, *P_cox‐4_* = 0.0005, *P_hxk‐1_* < 0.0001, *P_pyc‐1_* = 0.0064). (I) Quantified mitochondrial ROS on nematode head with MitoSOX probe after PdP NPs treatment in different doses (*n* = 3; *P*_0.0005_ > 0.9999, *P*_0.005_ = 0.9929, *P*_0.05_ = 0.9858, *P*_0.5_ = 0.9912). (J, K) Representative images and relatively quantified levels of mitochondrial ROS on nematode head with MitoSOX probe after H_2_O_2_ treatment in the absence or presence of preincubated PdP NPs. n_Control_ = 32, n_PdP NPs_ = 10, n_H2O2_ = 22, n_H2O2+PdP NPs_ = 27; *P* = 0.9843 (PdP NPs versus blank control), *P* <0.0001 (H_2_O_2_‐only control versus blank control), *P* = 0.0012 (H_2_O_2_‐treated PdP NPs group versus H_2_O_2_‐only control). Scale bar = 400 µm. Data represent means value ± standard errors. *P < 0.05, **P < 0.01, ***P < 0.001. The two‐sided one‐way ANOVA followed by a Tukey post hoc analysis (A, I, and K) or Sidak multiple comparisons test (D, E and H) was used for comparison among multiple groups; The two‐sided Student's *t*‐test was used for comparison between two groups (F,G).

The superior performances of PdP NPs on functionally mimicing the two crucial mitochondrial enzymes promoted us to further investigate its therapeutic potential to maintain the mitochondrial function in vivo environment. We first evaluated the biocompatibility of PdP NPs. Wildtype worms at L1 Larva stage were fed with PdP NPs for three days followed by measurements. We found that PdP NPs at different tested doses of 0.0005–0.5 µg mL^−1^ (tenfold increase) did not induce any adverse effect on nematodes’ survival rate, body length, and reproductive capacity (Figure 3A–C; Figure S5). C*c*O catalyzes the reduction of oxygen to water by receiving electrons from the heme‐containing cytochrome *c* (Cyt *c*) on mitochondrial respiration chain. Meanwhile the potential energy of electron converts into electrochemical gradient of the proton, which drives ATP production to support energy‐requiring reactions. We therefore detected oxygen consumption and ATP production to unambiguously testify the biological function of PdP NPs as nanozymes. As shown in Figure 3D–F, oxygen consumption rate (OCAR) and ATP showed an age‐dependent decrease in *C. elegans*. The basal and maximum oxygen consumption of young worms (adult day 1 and day 4) were not significantly affected by PdP NPs, whereas PdP NPs treatment induced significant upregulation in eld worms (adult day 7) by approximately 3.3‐ and 2.0‐fold compared to the control group, respectively (Figure 3D,E). Importantly, PdP NPs treatment also significantly up‐regulated ATP generation of *C. elegans* (Figure 3G; Figure S6). We further extended our characterization of the mitochondrial boosting activities of PdP NPs by including the mammalian cells. Compared to the control group, the ATP production was robustly up‐regulated by 56% and 15% in HEK293T and SH‐SY5Y cells, separately (Figures S7 and S8), implying the conserved capability of PdP NPs on promoting mitochondrial respiration chain function. On the basis of above results, we further explored whether PdP NPs could prevent mitochondrial damage under stressed environments. As shown in Figure S8, the fluorescence intensity of ATP in the SH‐SY5Y cells treated with either H_2_O_2_ or NaN_3_ (specific C*c*O inhibitor) was dramatically decreased. While the difference was partly reversed when PdP NPs were added. These results demonstrated that PdP NPs could effectively attenuate mitochondrial damage and relieve cellular energy scarcity caused by several mitochondrial toxins. To understand the possible influence on mitochondrial metabolism of PdP NPs, we also measured the influence of PdP NPs treatment on the expression of enzymes dominating key mitochondrial metabolic pathways. Notably, PdP NPs treatment demonstrated significant efficacy in up‐regulating the gene expression of enzymes controlling key metabolic pathways including the TCA cycle gene citrate synthase (*cts‐1*), the glycolysis genes hexokinase (*hxk‐1*), gluconeogenesis gene pyruvate carboxylase *(pyc‐1)* and cytochrome *c* oxidase IV (*cox‐4*), explaining that PdP NPs mainly boost mitochondrial metabolism of *C. elegans* (Figure 3H; Figure S9).

Next, we assessed the capability of PdP NPs to eliminate superoxide radicals, a key function of SOD toward stressed environment. 2’,7’‐dichlorodihydrofluorescein diacetate (H_2_DCFDA) and MitoSOX probes were used to label the intracellular and mitochondrial ROS, respectively. As shown in Figure 3I and Figures S10 and S11, the ROS level located at either mitochondrial or whole cell was not significantly upregulated by PdP NPs at tested doses relative to the control group, indicating its biocompatibility. When exposed to H_2_O_2_, the ROS level was significantly decreased in PdP NPs pretreated *C. elegans* (Figure 3J,K; Figure S11). It was worth mentioning that the effect of PdP NPs on reducing mitochondrial oxidative stress was also observed in SH‐SY5Y cells (Figure S12). This suggested that PdP NPs is an outstanding ROS scavenger that protect mitochondria from oxidative damage. Altogether, above studies indicated that PdP nanozymes are promising alternatives to C*c*O and SOD, and could well replicate their biological function in mitochondrial activation.

### 2.3 PdP NPs Treatment Maintain Mitochondrial Integrity and Act as an UPR^mt^ Activator to Refine Homeostasis

**FIGURE 4:**
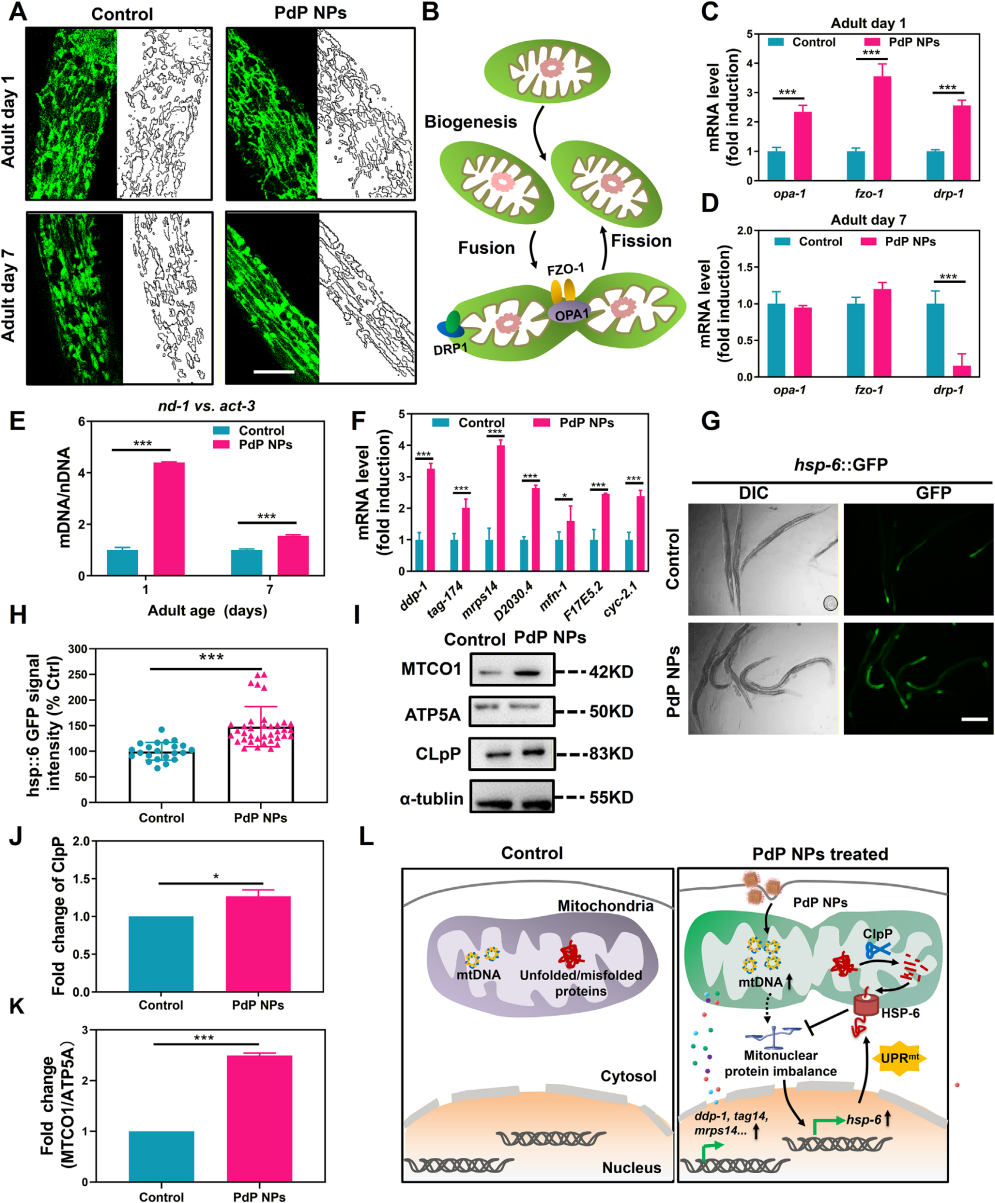
PdP NPs treatment improve mitochondrial homeostasis in *C. elegans*. (A) Representative images of the mitochondrial morphology in body wall muscle of transgenic *C. elegans* SJ4103 (zcIs17 [Pges‐1::GFPmt]) expressing pmyo‐3::mito::GFP reporter on adult day 1 and 7. Mitochondria in the fusion state shows tubular shape; mitochondria in the split state shows punctate shape which represents mitochondrial fragmentation (MF). Scale bar = 10 µm. (B) Schematic diagram of mitochondrial biogenesis and fusion/fission dynamics. (C) The mRNA level of mitochondrial homeostasis regulators at day 1 of adulthood (*n* = 3; *P_opa‐1_* < 0.0001, *P_fzo‐1_* < 0.0001, *P_drp‐1_* < 0.0001). (D) The mRNA level of mitochondrial homeostasis regulators at day 7 of adulthood (*n* = 3; *P_opa‐1_* = 0.9587, *P_fzo‐1_* = 0.2247, *P_drp‐1_* < 0.0001). (E) Mitochondrial biogenesis at day 1 and day 7 of adulthood, as evidenced by the increased mtDNA/nDNA ratio (*n* = 3). PdP NPs exposure induced a relative increase of mtDNA copy number in comparison to mock control (Left: *P* < 0.0001; Right: *P*< 0.0001). (F) Quantitation of nuclear genes encoding mitochondrial proteins by qRT‐PCR in N_2_ animals at day 1 of adulthood (*n* = 3; *P_ddp‐1_* < 0.0001, *P_tag‐174_* = 0.0002, *P_mrps14_* < 0.0001, *P_D2030.4_* < 0.0001,*P_mfn‐1_ = 0.0406*, *P_F17E5.2_* < 0.0001, *P_cyc‐2.1_* < 0.0001). Representative images and relatively quantified levels of HSP‐6::GFP were shown in panel G and H, separately (n_control_ = 22, n_PdP_ = 37; *P* < 0.0001). Scale bar = 200 µm. (I) Western blots pics of CLpP, MTCO1, SDHA and ATP5A in the absence or presence of preincubated PdP NPs. α‐tublin served as a loading control. (J) Quantitative analysis on the UPR^mt^ protease CLpP expression in *C. elegans* treated with PdP NPs (*P* = 0.047). (K) Quantitative analysis on the ratio of mtDNA‐encoded MTCO1 and nDNA‐encoded ATP5A in *C. elegans* treated with PdP NPs (*P* = 0.0006). (M) Schematic illustration of the effect of PdP NPs on mitochondrial homeostasis. The data was normalized by defining the mean value of control group as 100% and set 0 as 0%. *P < 0.05, **P < 0.01, ***P < 0.001. The two‐sided one‐way ANOVA followed by the Sidak multiple comparisons test was used for comparison among multiple groups and the two‐sided Student's *t*‐test was used for comparison between two groups.

The process of mitochondrial dysfunction is always accompanied by deregulation of mitochondrial homeostasis [13, 38]. Previous studies have proved that C*c*O and SOD are critically involved in mitochondrial dynamics and structural integrity [8, 9, 10]. Encouraged by the superior performances of PdP NPs as artificial nanozymes, we speculated that PdP NPs could support mitochondria to maintain integrity and homeostasis. To confirm the hypothesis, influence of PdP NPs on mitochondrial network's morphology was first monitored utilizing transgenic worms expressing mitochondria‐targeted pmyo‐3::mito::GFP in the muscle walls. Noticeably, compared to the young blank control worms, PdP NPs treated worms presented similar network pattern on day of adulthood 1 (Figure 4A; Figure S13). While when worms become old (on day of adulthood 7), exposure to PdP NPs moderately increased the mitochondrial network morphology with a shift from distinctive, circular‐shaped morphology characteristic of mitochondrial fragmentation to tubular structures (Figure 4A; Figure S13), implying that PdP NPs could maintain a healthier tubular mitochondrial network in eld animals. Mitochondrial fission depends upon dynamin‐related protein 1 (DRP‐1) while fusion depends upon FZO‐1 (ortholog of mitofusins MFN1 and MFN2, which are responsible for fusing the outer membrane) and OPA‐1 (ortholog of optical atrophy 1, which fuses the inner membrane) (Figure 4B). To understand the impact of PdP NPs on mitochondrial dynamics, the transcriptional expression of fission/fusion regulators *opa‐1, fzo‐1* and *drp‐1* was analyzed. As illustrated in Figure 4C,D, all these genes were up‐regulated in PdP NPs‐treated group on adult day 1, indicating that PdP NPs treatment was able to augment dynamics and stimulate self‐renewal of mitochondria during early days of young worms. While on adult day 7, *drp‐1* expression was down‐regulated whereas other genes returned to expression levels comparable to those of the control, implying that PdP NPs promote mitochondrial fusion of aging nematodes, which was in consistent with previous findings. Altogether, these data indicated that PdP NPs treatment is able to trigger a desirable shift of mitochondrial dynamics. In order to further confirm the protecting trend of PdP NPs on mitochondrial integrity, we also explored whether the nanozymes influence the mitochondrial abundance by evaluating the mtDNA/nDNA ratio. Excitingly, the mitochondrial contents consistently displayed up‐regulation trends on different tested days (Figure 4E; Figure S14). Ulteriorly, we verified the upregulation of mitochondrial‐function‐related genes by reverse transcription (RT)–qPCR analysis in worms (Figure 4F; Figure S15).

The unusual effect of PdP NPs inspired us to investigate further. The mitochondrial unfolded protein response (UPR^mt^) that maintains mitochondrial proteostasis is a conserved longevity mechanism in various animal models and a promising therapeutic target [24, 25]. Based on our observation, there was a sharp rise of UPR^mt^ biomarker‐HSP6 (the mammalian UPR^mt^ homolog Hsp60) expression when PdP NPs were introduced into the *C. elegans*, implying that PdP NPs had a promoting effect on UPR^mt^ (Figure 4G,H). Consistently, PdP NPs treatment induced upregulation of the quality control serine protease—caseinolytic protease proteolytic subunit (CLpP) at the protein level, indicative of therapeutic efficacy in UPR^mt^ induced proteostasis (Figure 4I,J). Above findings raise the question of how PdP NPs treatment induce the salutary effects upon activating UPR^mt^. Typically, mitochondrial proteins are encoded by both nuclear and mitochondrial genomes. An imbalance between the expression of proteins from these two sources could directly activate UPR^mt^, which could be verified by examining the expression of ATP synthase mitochondrial F1 complex subunit alpha (ATP5A) and succinate dehydrogenase complex subunit A (SDHA) encoded by nuclear DNA, as well as mitochondrially encoded cytochrome *c* oxidase I (MTCO1) encoded by mitochondrial DNA. Remarkably, western blot results identified an imbalance between mitochondria versus nuclear proteins in PdP NPs‐treated group compared to the mock treated group (Figure 4I,K; Figure S16), which we speculated was due to the improved biogenesis induced by PdP NPs treatment. Altogether, above observations revealed the capacity of PdP NPs on refining mitochondrial homeostasis and maintaining integrity, which implied the therapeutic potential of PdP NPs in promoting physical fitness and relieving aging‐related microenvironment disorders (Figure 4L).

### 2.4 PdP NPs Treatment Prolonged Lifespan and Resist Aging

**FIGURE 5:**
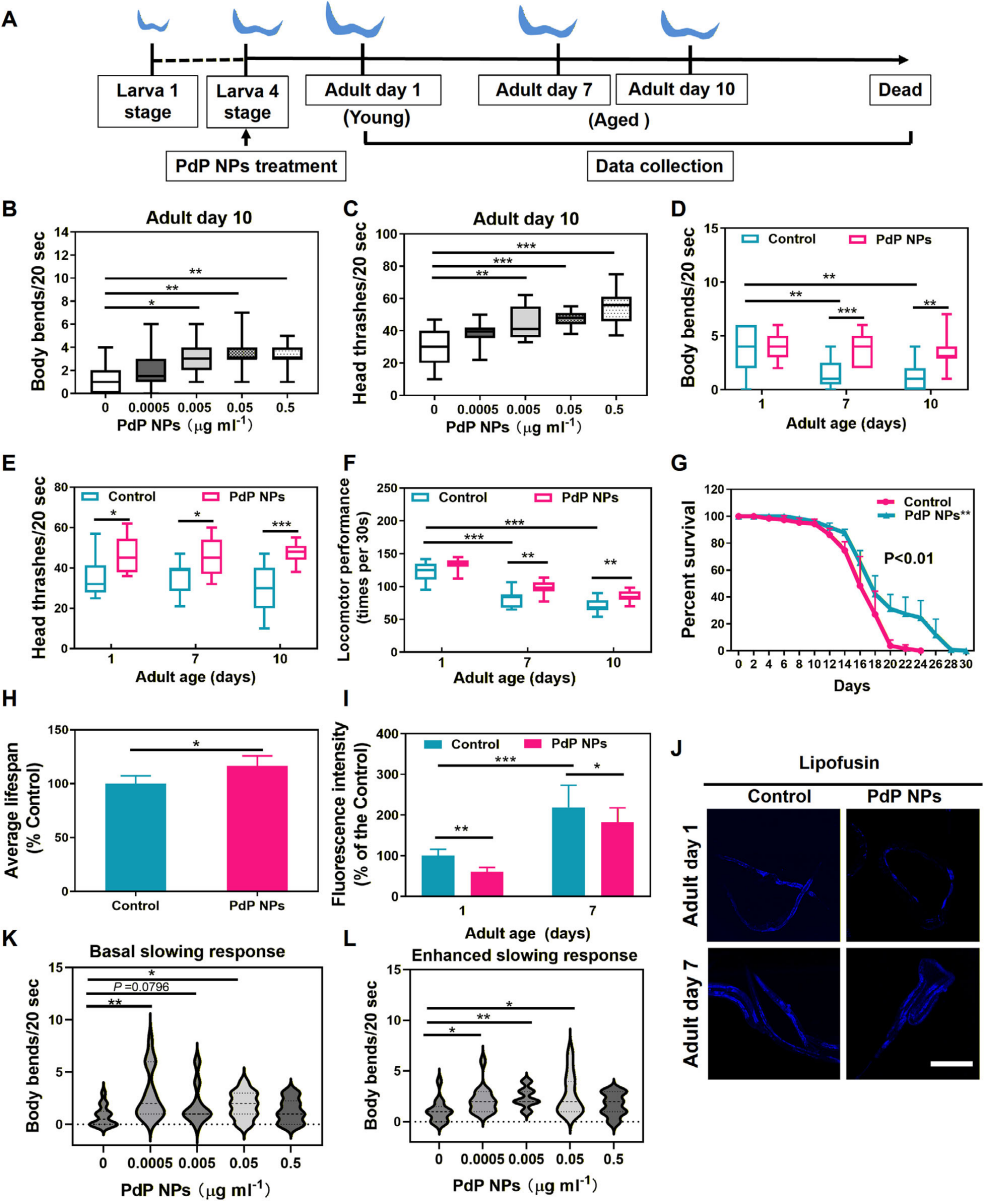
PdP NPs treatment promote extension of healthy lifespan in *C. elegans*. (A) Schematic diagram of the anti‐aging experiment procedure. (B) Body bends of worms on adult day 10 pretreated with different concentrations of PdP NPs (*n* = 12–15; *P*_0.0005_ = 0.6994, *P*_0.005_ = 0.0102, *P*_0.05_ = 0.0023, *P*_0.5_ = 0.0064). (C) Head thrashes of worms on adult day 10 pretreated with different concentrations of PdP NPs (*n* = 10–15; *P*_0.0005_ = 0.1246, *P*_0.005_ = 0.0023, *P*_0.05_ = 0.0003, *P*_0.5_ < 0.0001). (D) Body bends of young (adult day 1), middle old‐age (adult day 7) and old (adult day 10) worms were measured (*n* = 11–18). *P* = 0.0046 (AD7 control versus AD1 control); *P* = 0.0021 (AD10 control versus AD1 control); *P* = 0.7287 (AD1 PdP NPs versus AD1 control); *P* = 0.0009 (AD7 PdP NPs versus AD7 control); *P* = 0.0024 (AD10 PdP NPs versus AD10 control). (E) Head thrashes of young (adult day 1), middle old‐age (adult day 7) and old (adult day 10) worms were tested (*n* = 10–15). *P* = 0.0314 (AD1 PdP NPs versus AD1 control); *P* = 0.05 (AD7 PdP NPs versus AD7 control); *P* < 0.0001 (AD10 PdP NPs versus AD10 control). (F) Locomotion performance of young (adult day 1), middle old‐age (adult day 7) and old (adult day 10) worms were tested (*n* = 13‐18). *P* < 0.0001 (AD7 control versus AD1 control); *P* < 0.0001 (AD10 control versus AD1 control); *P* = 0.1658 (AD1 PdP NPs versus AD1 control); *P* = 0.0027 (AD7 PdP NPs versus AD7 control); *P* = 0.0061 (AD10 PdP NPs versus AD10 control). (G) Lifespan curves of worms in the presence of PdP NPs compared to untreated group (n_control_ = 4, n_PdP_ =3; *P =* 0.0033). (H) Average lifespan of worms were measured (n_control_ = 204, n_PdP_ = 216; *P <* 0.0001). (I, J) Quantification and representative pictures of nematode lipofuscin fluorescence were detected by fluorescence microscope (Left, n_control_ = 13, n_PdP_ = 16, *P* = 0.0067; Right, n_control_ = 14, n_PdP_ = 9, *P* = 0.0282). Scale bar = 0.5 mm. (K) Quantified basal slowing response (BSR) and enhanced slowing response (ESR) of middle old‐age worms (adult day 7) indicating locomotor activity under harsh environment (*n*_0_ = 14, n_0.0005_ = 15, n_0.005_ = 9, n_0.05_ = 12, n_0.5_ = 10; *P*_0.0005_ = 0.0057, *P*_0.005_ = 0.0796, *P*_0.05_ = 0.0256, *P*_0.5_ = 0.2514); (L) Quantified enhanced slowing response (ESR) of middle old‐age worms (adult day 7) indicating locomotor activity under harsh environment (n_0_ = 13, n_0.0005_ = 14, n_0.005_ = 12, n_0.05_ = 15, n_0.5_ = 13; *P*_0.0005_ = 0.0353, *P*_0.005_ = 0.0044, *P*_0.05_ = 0.0142, *P*_0.5_ = 0.1712). For BSR, nematodes were incubated with E. coli OP50 at 35°C for 3 h; For ESR, nematodes were first incubated at 35°C for 3 h, then deprived of food for 30 min. The body bends were analyzed subsequently. Data represent means value ± standard errors. In panels of G‐I, the data was normalized by defining the mean value of control group as 100% and set 0 as 0%.*P < 0.05, **P < 0.01, ***P < 0.001. The two‐sided one‐way ANOVA followed by a Tukey post hoc analysis (B,C, K, and L) or Sidak multiple comparisons test (D, E, F, and I) was used for comparison among multiple groups. The two‐sided Student's *t*‐test was used for comparison between two groups (G, H).

Encouraged by these superior performances of PdP NPs on improving mitochondrial function and promoting homeostasis, therapeutic effects of PdP NPs on prolonging the healthy lifespan were investigated at *C. elegans* level. As depicted in Figure 5A, synthesized worms were treated with PdP NPs with diets since L4 larva stage until death. During the aging process, living organisms undergo several degenerative functional and structural changes. Delaying or reversing these degenerative changes is critical to prolong the healthy longevity. In order to examine the anti‐aging potential of PdP NPs, we first evaluated the impact of nanozymes on worm behavioral deterioration along with aging. According to the observations, PdP NPs supplement almost had no effect on the body behavior of young worms (Figure S17). In contrast, obvious improvement in aged wild‐type animals was observed after PdP NPs therapy in a dose‐dependent mode (Figure 5B–E; Figure S18). The pharynx is an important organ composed of pharyngeal muscles that shows age‐dependent loss of function and structure. We therefore also measured pharynx locomotion to investigate the health of the rescued animals by PdP NPs. Consistent with above findings, PdP NPs treatment almost had no effect in young worms while exhibited a significant improvement in the locomotor performance along with aging (Figure 5F). In all, above findings implied the therapeutic potential of PdP NPs in decelerating the aging‐related behavioral decline.

To reveal the anti‐aging activity of PdP NPs more directly, we continued to explore the impact of nanozymes on the lifespan. Excitingly, the average lifespan extension (∼19.7%) was observed in PdP NPs‐treated group and the longest survival time of nematodes was extended up to 30 days compared to 24 days in control group (Figure 5G,H). Additionally, we quantified aging biomarker on different life developmental stages as a next step toward validating the anti‐aging effect of PdP NPs. Lipofuscin is a readily observable biomarker of aging, and its fluorescence intensity indicates physiological aging status in nematodes. Confocal laser scanning microscopy (CLSM) images and quantitative analysis showed that the accumulated lipofuscin in *C. elegans* on adult day 1 and day 7 was significantly decreased by 39.6% and 16.8% in PdP NPs‐treated group compared to the control group, separately (Figure 5I,J). To further confirm that the anti‐aging effect was a direct consequence of PdP NPs treatment, we also evaluated the impact of several other nanoparticles (Prussian NPs, CeO_2_ NPs, MnO_2_ NPs) on the athletic capability and lipofuscin accumulation. None of these nanomaterials exerted significant effects at the concentrations tested (Figures S19 and S20). These results implied a unique anti‐aging property of PdP NPs. To rule out PdP NPs‐mediated alteration of microbial metabolism, we also fed worms with heat‐killed OP50 bacteria as nematodes’ food source. Notably, PdP NPs treatment still conferred a significant anti‐aging phenotype—evidenced by reduced enhanced locomotor performance (Figure S20). Furthermore, PdP NPs at gradient concentrations exerted no significant effect on the growth rate of live OP50 bacteria (Figure S21). Collectively, these observations rule out perturbation of bacterial growth or metabolism as the mechanism underlying the lifespan extension elicited by PdP NPs in *C. elegans*. The long‐lived nematodes normally showed stress‐tolerant potential. To assess the anti‐stress potential of PdP NPs, its efficacy to protect worms against harsh conditions was also verified. In comparison with control group, PdP NPs pretreated worms exhibited significantly stronger athletic capability in high temperature conditions (Figure 5K,L; Figure S22). In essence, above results strongly support that PdP NPs could not only effectively extend healthy lifespan of *C. elegans*, but also provide protections against harsh environment.

Additionally, in order to further understand the role of UPR^mt^ in PdP NPs ‐mediated anti‐aging effects, we also studied the influence of PdP NPs upon mutant *C. elegans (fx0259)* with loss‐of‐function of *dev‐1*, which is the essential transcription factors in UPR^mt^ pathway. Interestingly, the anti‐aging effect by supplementation of PdP NPs was partly prevented in the *dve‐1* mutant, highlighting the importance of UPR^mt^ in PdP NPs ‐mediated anti‐aging effects (Figure S23).

### 2.5 PdP NPs Treatment Rescues Neuron Death and Behavior Disorder of Huntington *C. elegans*

**FIGURE 6:**
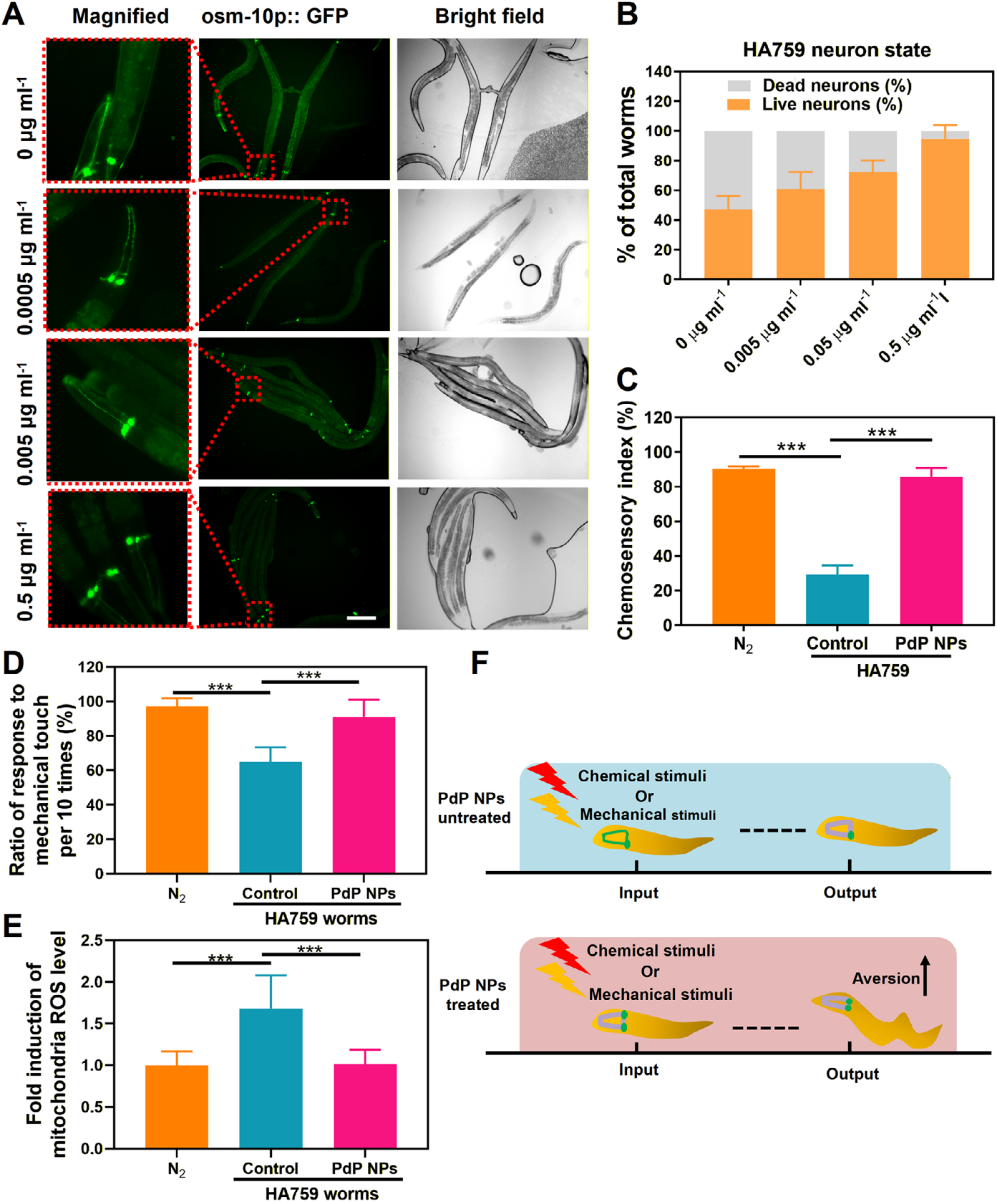
Neuronal protection effect of PdP NPs in Huntington *C. elegans*. (A) Representative live fluorescence imaging of ASH neurons in transgenic *C. elegans* strain HA759 with huntingtin fragments Htt‐Q150 highly expressed in ASH sensory neurons. Scale bar = 0.5 mm. (B) Survival rate of ASH neurons of transgenic HA759 after PdP NPs treatment (n_0_ = 28; n_0.005_ *=* 24; n_0.05_ *=* 24; n_0.5_ *=* 22). Death of ASH neurons was defined by lack of bilateral GFP fluorescence. (C) Effect of PdP NPs on chemosensory behavior assay in *C. elegans*. n_N2 control_ = 276, n_HA759 Control_ = 250, n_HA759 PdP NPs_ = 210; *P* < 0.0001 (HA759 control versus N_2_ control); *P* < 0.0001 (HA759 PdP NPs versus HA759 control). (D) Effect of PdP NPs on mechanical sensory behavior in *C. elegans* (*n =* 10). *P* < 0.0001 (HA759 control versus N_2_ control); *P* < 0.0001 (HA759 PdP NPs versus HA759 control). (E) Relatively quantified levels of mitochondrial ROS on N_2_ and HA759 nematode head in the absence or presence of preincubated PdP NPs (n_N2 control_ = 36, n_HA759 control_ = 49, n_HA759 PdP NPs_ = 30; *P* < 0.0001 (HA759 control versus N_2_ control), *P* < 0.0001 (HA759 PdP NPs versus HA759 control). (F) Schematic diagram of the PdP NPs’ protective effects on behavioral dysfunction in HA759 nematodes. Data represent means value ± standard errors. *P < 0.05, **P < 0.01, ***P < 0.001. The two‐sided one‐way ANOVA followed by a Tukey post hoc analysis was used for comparison among multiple groups.

Mitochondrial dysfunction has been implicated in the pathogenesis of neurodegenerative disease—an example of unhealthy ageing of the brain. To figure out the potential protecting mechanism of PdP NPs against neurodegenerative disorders, transgenic Huntington *C. elegans* strain HA759, which highly expressed huntingtin fragments Htt‐Q150 (a polyQ150 tract derived from human Huntington) in ASH neuron, was adopted to probe its effect on neural viability and function. We treated HA759 strain with PdP NPs since the early L1 stage, during which the nervous system is the most sensitive and vulnerable to detrimental effects among the whole life [39]. The survival rate of ASH neurons was first evaluated by the foci of GFP signals. According to the results, the worms fed with PdP NPs showed higher neuronal survival rate in a dose‐dependent mode, which was approximately 1.29 times (with 0.005 µg mL^−1^ PdP NPs), 1.53 times (with 0.05 µg mL^−1^ PdP NPs) and 2.0 times (with 0.5 µg mL^−1^ PdP NPs) than that of controls, respectively (Figure 6A,B). The ASH neuron is required for the perception of pain, mechanical stimuli, environmental osmotic pressure and chemical factors, which promote the avoidance response of worms against harmful stimuli. When these neurons are eliminated, avoidance behavior of *C. elegans* is disrupted. To further probe the therapeutic roles of PdP NPs on neuronal dysfunction, sensory and mechanical responses assays were adopted (Figure S24). As shown in Figure 6C, compared with N_2_ wild‐type (chemosensory index: ∼0.90), the majority of transgenic HA759 nematodes lost the ability to sense noxious stimuli due to ASH neuronal death with a chemosensory index of ∼0.29. Notably, PdP NPs dramatically improved functions of ASH neurons, with the chemosensory index increased up to ∼0.85. Meanwhile, mechanosensory measurement also confirmed the potential of PdP NPs on reducing neuronal damage. In contrast to wide‐type N_2_ nematodes, only 45% HA759 *C. elegans* normally responded to physical touch (Figure 6D). While in PdP NPs pretreated HA759 nematodes, the percentage reached 81% with normal response to mechanical disturbance (Figure 6D), illustrating that PdP NPs could improve the avoidance response of ASH neurons against harmful stimuli. To continuously figure out the beneficial effect exerted by PdP NPs, we explored the potential interference of mitochondrial ROS level by PdP NPs treatment. Remarkably, MitoSOX staining revealed that transgenetic huntington *C. elegans* strain (HA759) showed higher ROS level compared to its wildtype control. While PdP NPs significantly reduced the ROS content and alleviated the mitochondrial stress (Figure 6E; Figure S25). Taken together, all these observations demonstrated that PdP NPs treatment could prevent neurons from death and provide protective effects against neuronal dysfunction (Figure 6F).

## 3 Conclusion

In summary, we presented simply constructed PdP NPs that functionally mimic crucial mitochondrial enzymes (C*c*O and SOD) that enable efficient activation of mitochondrial organelle without any stimulus for catalytic therapy against aging and aging‐related neurodegenerative disorders. Benefiting from the ultrasmall size and superior catalytic activity, the non‐toxic PdP NPs were shown to significantly boost mitochondrial metabolism and enable the sufficient generation of ATP to relieve cellular energy scarcity of the aging *C. elegans* individuals while decrease mitochondrial oxidative stress. Further experiments further validated the protective effects of PdP NPs in mammalian cell models of mitochondrial dysfunction. At the same time, our study showed that dietary low level of PdP NPs could not only refine mitochondrial dynamics, but also promote mitochondrial biogenesis and activate mitochondrial proteostasis sensor UPR^mt^, therefore revealed as important mitochondrial homeostasis regulators. More importantly, proved by comprehensive in vivo experiments leveraging *C. elegans*, PdP NPs treatment remarkably extended lifespan by 19.7 percent and enhanced anti‐aging therapeutic efficacy. Furthermore, the PdP NPs therapy exhibited highly efficient protection to neuron and allowed for alleviation of neurodegenerative disorders in the Huntington *C. elegans* model. This work is envisioned to provide a proof of concept for the use of nanozymes that mimic mitochondrial enzymes to rewire mitochondrial metabolism and expand the potential of bioactive palladium‐based nanomaterials as mitochondrial stimulators in battling aging and neurodegeneration. Despite these encouraging findings, this study still has limitations: it remains at the cellular and invertebrate levels, and the pharmacokinetics, long‐term biosafety, and therapeutic efficacy of PdP NPs in mammalian animals require further clarification to advance clinical translation.

## 4 Experimental Section

### 4.1 Preparation and Characterization of PdP NPs

The Pd‐PVP nanoparticles (PdP NPs) were synthesized by the reduction of the Pd ions according to a previously reported method [40, 41]. Typically, the synthesis of the Pd NPs was performed at 60°C using an aqueous solution of PdCl_2_ with PVP as a reducing and stabilizing agent. The palladium precursor solution (H_2_PdCl_4_) was prepared by heating PdCl_2_, doubly deionized water and HCl. Then a solution containing H_2_PdCl_4_, PVP, HCl and doubly deionized water was heated. When the solution began to reflux, ethanol was added. The solution was then refluxed for 3 h, and this resulted in a dark brown colloidal Pd solution. The reduction of PdCl_2_ to Pd0 by PVP prevents the aggregation of nanoparticles through the repulsive forces that emerge from the hydrophobic carbon chains; thus, the PdP NPs were formed. The PVP–Pd interactions are stabilized by the donation of a single pair of electrons from the nitrogen or carbonyl oxygen of PVP to the Pd ion.

### 4.2 Reagents and Strains

ATP determination kit (A22066) and MitoSOX were purchased from Thermo Fisher. BCA assay kit and DCFH‐DA were purchased from Beyotime. TRIZOL and M‐MLV Reverse Transcriptase were purchased from Promega. NaN_3_ was used as an anesthetic for worms or as a C*c*O inhibitor in cellular experiments. Wild‐type strain, transgenetic worm strain SJ4100 (zcIs13 [HSP‐6::GFP]) and HA759 {rtIs11[osm‐10p::GFP+osm‐10p::HtnQ150+Dpy‐20(+)]} worm strain were provided by the Caenorhabditis Genetics Center (University of Minnesota). Transgenetic worm strain SJ4103 (zcIs14 [myo‐3::GFP (mit)]) was a gift from Dr Chen Liangwen (Huainan Normal University). FX0259 (*dve‐1*) mutant worms were generally granted by Professor Xiaochen Wang (Institute of Biophisics, Chinese Academy of Sciences). We employed standard nematode culture conditions. All strains were maintained at 20°C on Nematode Growth Media agar supplemented with Escherichia coli (OP50). Unless otherwise stated, L4 stage synchronized *C. elegans* were transferred to NGM plates containing 0.05 ug mL^−1^ concentrations of PdP NPs until day of analysis, which was performed with the investigator blinded to sample identities. Pyrimidine analogue 5‐fluoro‐2′‐deoxyuridine (FUdR, Sigma; St. Louis) was supplemented with a concentration of 50 µM to L4 stage worms to prevent the development of progeny.

### 4.3 Characterization of PdP NPs

The morphology of PdP NPs were characterized by transmission electron microscopy (TEM) and high‐magnification TEM. The size distribution and zeta potential of the nanoparticles was measured by a dynamic light scattering equipment by Zetasizer Nano‐ZS from Malvern Instruments. The crystal form of PdP NPs was measured by XPS (Thermo Scientific K‐Alpha) and EDX (OXFORD instrument).

### 4.4 Preparation of Ferrous Cytochrome *c*

Horse heart cytochrome c (Cyt *c*) was obtained from Sigma Aldrich. Ferrous Cyt *c* was prepared by adding a small amount of sodium hyposulphite (Na_2_S_2_O_4_) to the solution of ferric Cyt *c*. After reduction, the Na_2_S_2_O_4_ was removed by being passed through a Sephadex G‐25 PD‐10 desalting column (GE Healthcare) equilibrated with 100 mM phosphate buffer (pH 7.2) [42].

### 4.5 C*c*O‐Like Activity of PdP NPs

Cyt *c* transfers electrons by undergoing oxidation and reduction of the iron cation (which can be alternately 2t or 3t) in Cyt *c*’s heme moiety. Therefore, the additional peak at 550 nm can, therefore, be monitored spectroscopically to quantitate the amount of reduced Cyt *c* [43]. In this study, the Cyt *c* oxidase (C*c*O) activity was monitored by measuring the decrease in the absorbance of reduced Cyt *c* solution at 414nm and 550 nm spectrophotometrically with a UV–vis multimode microplate reader (Tecan Spark) operating under time‐drive mode. The reaction mixture contained PdP NPs and Cyt *c* (0.2 µg mL^−1^) in phosphate buffer (0 mM, pH 7.0) at 25°C. The kinetic analysis was carried out by varying the concentration of PdP NPs (0.1–100 µg mL^−1^) or incubation time (0–30 min) at a constant concentration of Cyt *c*. The auto‐oxidation of Cyt *c* without the addition of PdP NPs was performed with the same procedure as stated above, except that PdP NPs suspension was not added.

### 4.6 SOD‐Like Activity of PdP NPs

SOD is able to suppress the reaction of 2‐(2‐methoxy‐4‐nitrophenyl)‐3‐(4‐nitrophenyl)‐5‐(2,4‐disulfophenyl)‐2H‐tetrazolium (WST‐8) and oxygen produced by xanthine oxidase, which could reduce the production of water‐soluble formazan. Therefore, SOD activity is negatively correlated with the amount of formazan. In the study, SOD‐like activity of PdP NPs was studied spectrophotometrically according to the manufacturer's instructions of SOD enzyme activity detection kit (Cat.no. S0101S, Beyotime Biotechnology). To verify the $O_{2}^{\cdot -}$ scavenging ability of PdP NPs, WST‐8, xanthine, and xanthine oxidase were mixed in PdP NPs dispersions at distinct concentrations (0, 0.1, 0.2, 1, 10, 50, and 100 µg mL ^−1^). The absorption changes of water‐soluble formazan were then monitored at 450 nm using multimode microplate reader (Tecan Spark).

### 4.7 Determination of Partially Reduced Oxygen Species (PROS) Generation

The generation of PROS in the C*c*O‐like reaction was determined by using multiple assays.

#### 4.7.1 Superoxide (O_2_^·−^) Detection by WST‐1 Assay

Superoxide production was measured using the tetrazolium salt WST‐1 (2‐(4‐iodophenyl)‐3‐(4‐nitrophenyl)‐5‐(2,4‐disulfophenyl)‐2H‐tetrazolium, monosodium salt). Upon reaction with $O_{2}^{\cdot -}$, WST‐1 is reduced to formazan, which exhibits a characteristic absorption maximum at 440 nm. The amount of formazan produced in the reaction mixture corresponds directly with superoxide levels. The reaction solution contained WST‐1, PdP nanozymes (0.1, 1, 10 µg mL^−1^) and Cyt *c* (10 µM mL^−1^) in phosphate buffer (50 mM, pH 7.4) at 25°C in the presence of WST‐1 and the absorbance was determined at 440 nm [32].

#### 4.7.2 Detection of Hydrogen Peroxide (H_2_O_2_) by Amplex Red Assay

To rule out potential H_2_O_2_ generation, an Amplex Red assay was performed. In this assay, Amplex Red reacts with H_2_O_2_ in the presence of horseradish peroxidase (HRP), producing the fluorescent compound resorufin, which exhibits excitation and emission maxima at 563 nm and 587 nm, respectively. Thus, H_2_O_2_ production during the nanoparticle–cytochrome *c* interaction can be monitored fluorometrically using the Amplex Red–HRP‐coupled system [32]. The reaction mixture contained: PdP NPs (0.1, 1, and 10 µg mL^−1^), Cyt *c* (10 µM), HRP (0.5 mg/mL), Amplex Red (10 µM) in 50 mM phosphate buffer (pH 7.4). Reactions were conducted at 25°C.

### 4.8 Cell Culture

HEK293T cells and SH‐SY5Y cells were cultured in the Dulbecco's modified Eagle Medium (HyClone). All the media contained 10% fetal bovine serum (Gibco) and 1% antibiotics (penicillin/streptomycin), and all cells were cultured at 37°C in a 5% CO_2_ atmosphere.

### 4.9 Egg‐Laying Rate and Body Length

Synchronized L1‐stage larvae were cultured on NGM agar plates seeded with E. coli OP50 and supplemented with PdP NPs (0.005–0.5 µg mL^−^^1^) at 20°C. Each day, a fraction of the synchronized animals at indicated developmental age were randomly selected and imaged under microscope. Body length was quantified by tracing the full‐body outline using ImageJ software. After 3 days of exposure, approximately 30 nematodes from tested groups were transferred to fresh NGM agar plates and allowed to lay eggs for 2 h. The egg‐laying rate was calculated as the average number of eggs laid per hour by 10 nematodes during this spawning period.

### 4.10 ATP Determination

The relative ATP content was measured using ATP determination kit [25]. The assay was performed with the investigator blinded to sample identities.

For nematodes experiments, approximately 100 age‐synchronized L4‐stage nematodes were placed on OP50 plates with FUDR (50 µM) and PdP NPs (0.05 µg mL^−1^) at 20°C until adult day 1 and day 7. The nematodes were collected and washed three times. Worm pellets were treated with three freeze‐thaw cycles for 5 times, then boiled for 15 min to releases ATP and destroy ATPase activity; Samples were then spun at 4°C at 11000 g for 10 min. The diluted supernatants were used for ATP measurement according to the manufacturer's instructions [25]. For normalization, protein levels from the same preparation were determined using a BCA assay.

For cells test, SH‐SY5Y or HEK293T cells were seeded on 12‐well plates at a density of 5 × 10^4^–1 × 10^5^ cells per well and cultured for 24 h. Cells were then treated with PdP NPs (0.5 µg/mL) for 12 h. Subsequently, culture medium was replaced with DMEM containing either 2.5 mM sodium azide (NaN_3_), 100 µM hydrogen peroxide (H_2_O_2_), or vehicle control, and incubated for an additional 1.5 h. Then the cells were washed with PBS and collected to destroy ATPase activity. Samples were centrifuged at 4°C at 11000 g for 10 min. The diluted supernatants were analyzed for ATP content following the manufacturer's instructions [25]. Total protein concentration was determined via BCA assay for data normalization.

### 4.11 Oxygen Consumption Measurement

Basal oxygen consumption was measured per previous methods using a Seahorse XF24 equipment (Seahorse Bioscience). In brief, L4 staged synchronized nematodes per group were transferred to designated drug plates, followed by OCR detection on adult days 1, 4 or 7. On the day of the experiment, all the nematodes were collected and transferred to 24‐well standard Seahorse assay plate within 0.5 mL M9 buffer per well, and basal/maximum oxygen consumption rate was measured six times and normalized to the number of worms each well.

### 4.12 Measurement of Reactive Oxygen Species (ROS)

Oxidative stress by H_2_O_2_ as stressor was induced in *C. elegans* and SH‐SY5Y cells to test the anti‐oxidative efficacy of PdP NPs on mitochondria.

For *C. elegans*, the L4 stage synchronized *C. elegans* were pretreated with FUDR (50 µM) and PdP NPs (0.05 µg mL^−1^) as previously described for two days. Then collected nematodes were exposed to 50 µM H_2_O_2_ for 30 min, followed by being washed in ice‐cold M9 buffer for 5 times. For whole‐cell ROS quantification, nematodes were stained with ROS probe DCFH‐DA (Molecular Probes, 25 µM) for 30 min at room temperature; For mitochondrial ROS quantification, nematodes were incubated with MitoSOX Red (Invitrogen/ThermoFisher, M36008, 1:200 MitoSox stock solution) for 20 min at room temperature. Then nematodes were washed for 5 times in ice‐cold M9 buffer to eliminate the DCFH‐DA/MitoSox reagent. Finally, the worms were transferred to agarose pads for assays under a fluorescence microscope. The whole‐cell ROS quantification was done by Image J software using green fluorescent signal located at the worms’ whole body; The mitochondrial ROS quantification was done by Image J software using red fluorescent signal located at the worms’ head.

For SH‐SY5Y cells, 5 × 10^3^–1 × 10^4^ cells per well were seeded in 96‐well plates and cultured for 24 h. Subsequently, the cells were incubated with 0.05 or 0.5 µg mL^−1^ PdP NPs for 12 h. Thereafter, oxidative stress was induced by adding Dulbecco's Modified Eagle Medium (DMEM) containing 100 µM H_2_O_2_ (or vehicle control) for 1.5 h. Then cells were washed once with phosphate‐buffered saline (PBS), followed by incubation with 2.5 µM MitoSOX Red in phenol red–free DMEM at 37°C for 20 min. Finally, cells were washed using PBS to eliminate residual MitoSox reagent, and fluorescence intensity was measured using Tecan Spark multimode microplate reader (excitation: 510 nm; emission: 580 nm).

### 4.13 *C. elegans* Behavioral Assays

Synchronized L4 stage worms were placed on OP50 plates with FUDR (50 µM) and PdP NPs at indicated doses at 20°C until the tested days. Pharyngeal pumping rates were measured by counting the number of contractions in the pharyngeal terminal bulb per 30 s of worms on the bacterial lawn under a dissection scope; Body bend rate was assessed under a dissection microscope by recording and counting the number of body bends (changes in the body bend at the mid‐body point per 20s) for each worm on solid agar plates without bacterial lawn; The head thrashing of the worms was counted using a hand‐held counter under a dissection scope on solid agar plates without bacterial lawn. All above behavioral assays were performed at least 10 individual worms per tested group on indicated nematode age, which is 1, 7 and 10 days from adulthood.

The basal slowing response (BSR) and enhanced slowing response (ESR) of worms were measured as described previously [25]. For BSR, we incubated the worms at 35‐mm NGM plates seeded with E. coli OP50 at 35°C for 3 h, and then allowed the plates to cool down to room temperature. For ESR, we incubated the worms at 35‐mm NGM plates seeded with E. coli OP50 at 35°C for 3 h, then deprived of food for 30 min. Then, the body bends were analyzed according to the procedures introduced above.

### 4.14 Analysis of Mitochondrial Morphology in Body‐Wall Muscle Cells

Synchronized L4 stage worms were placed on OP50 plates with FUDR (50 µM) and PdP NPs (0.05 µg mL^−1^) at 20°C until the tested days. SJ4103 carrying the mitochondrial matrix reporter that was composed of the myo‐3 promoter ligated to a mitochondrial leader sequence fused to GFP (referred to as “mitoGFP”) were utilized. *C. elegans* collected were placed on 2% agarose pads and anesthetized with 20 mM NaN_3_ before fluorescence observation (excitation: 488 nm; emission: 507 nm) under a fluorescence microscope. Mitochondrial morphology in nematodes was measured by examining a consistent region near the vulva. The morphology was considered normal when the majority of the cells within a single worm had ordered, tubular‐shaped mitochondria. Fragmented cells consisted of disorganized circular forms of mitochondria and fused mitochondria had elongated and connected formations. All worm slides were freshly constructed and imaged within 15 min to avoid side effect of NaN_3_ on autophagy. Images of about 15 worms in each group of the experiment were collected and analyzed using Fiji image J software.

### 4.15 Lifespan Assay on Solid Plates

Lifespan assays were performed at 20°C as previously mentioned [26, 44]. Worms were synchronized by NaOH and hypochlorite solution. The synchronized L4 population of worms were randomly split to respective condition plate in a density of about 30–80 worms per plate dish. PdP NPs and FuDR mixed with OP50 were added to the NGM media with final concentrations of 0.05 µg mL^−1^ and 50 µM, separately. The first day of adulthood was considered day 0. Adults were scored manually as dead or alive every other day. A worm was scored as dead when not responding to three repeated proddings. Worms that crawled off the plate, ruptured, or died from internal hatching were excluded from the analysis. Survival curve was plotted using GraphPad Prism 9.

### 4.16 Lipofuscin Measurement

Autofluorescent lipofuscin intensity was measured as described [26, 44]. In brief, synchronized L4 stage worms were placed on OP50 plates with FUDR (50 µM) and PdP NPs (0.05 µg mL^−1^) at 20°C until the tested days. Then the collected animals were anesthetized and arranged on an agarose pad. The lipofusin was assayed using a fluorescence microscope at 365 nm excitation and 420 nm emission wavelengths. All worm slides were freshly constructed and imaged within 15 min to avoid side effect of NaN_3_ on autophagy. Images of about 10–15 worms in each group of the experiment were collected and analyzed using Fiji image J software.

### 4.17 Chemotaxis Assay

Animal's sensory neurons functionality were assessed by quantifying repulsion to glycerol as previously described [45]. A timed egg‐lay was performed to synchronize populations. The synchronized L1‐stages HA759 nematodes were pretreated with or without PdP NPs (0.05 µg mL^−1^) for three days. Then the collected worms were washed three times with M9 buffer to remove bacteria. Glycerol was spread along the midline of the plate to divide the NGM plate into identical regions (A and B). When glycerol infiltrated the chemotaxis agar, 1% butanedione (2 µL) and 200 mM NaN_3_ (2 µL) were added to the plate A surface point, then the worms were placed on B‐region. Afterward, the NGM plate was placed in a 23‐degree incubator for 90 min. The number of nematodes in areas A and B was counted, and the chemosensory index was calculated as B/ (A + B).

### 4.18 Mechanical Stimulation Assay

The mechanical stimulation sensitivity was evaluated using a standardized mechanical stimulation assay [45]. Synchronized L1‐stage HA759 worms were treated with or without PdP NPs (0.05 µg mL^−1^) for three days. Individual nematodes were subjected to 10 sequential tactile stimuli delivered to the head region using a fine platinum wire, and the response ratio was calculated as the proportion of trials eliciting an avoidance reaction. Statistical analysis was performed to compare response frequencies between experimental groups.

### 4.19 qPCR for mRNA Level Assay

Synchronous L4 stage *C. elegans* were treated with FUDR (50 µM) and PdP NPs (0.05 µg mL^−1^) until adult day 1 or adult day 7. At the end of the treatment, nematodes were collected and washed once in ice‐cold PBS buffer, followed by lysis with Trizol (Invitrogen). After that, 2 µg of RNA was subjected to reverse transcription into cDNA by HiScript II SuperMix for qPCR (Cat. no. R223‐01, Vazyme Biotech Co., Ltd.). Then RT‐PCR reactions were prepared using PowerUp SYBR Green Master mix (Thermo Fisher Scientific, A25742) and performed on a Thermal cycler (Bio‐Rad) machine. All data were analyzed using the ΔΔCq method and normalized to the control using *actin‐1* as internal control. Absolute quantification of the mtDNA copy number of nemotodes was conducted by Q‐PCR as previously described [26, 44]. The ratio of relative gene expression values for *nd‐1* versus *actin‐3* represents mtDNA per nuclear genome. The results were confirmed with a second mitochondrial gene *mtce.26* versus *actin‐3*. The primers are shown in Table S1.

### 4.20 Western Blot Tests

The *C. elegans* were pretreated with PdP NPs as previously described. Then the worms were harvested and washed three times with M9 buffer for three times. Samples were then lysed in the RIPA lysis buffer supplemented with 1 µM PMSF and phosphatase inhibitors (ThermoFisher, 78446). Samples were sonicated for 5 min with intermittent ice incubation. The collected proteins were separated on SDS‐PAGE gels and revealed by western blot using anti‐ATP5A (Abcam), anti‐MTCO1 (Abcam), anti‐CLpP (Proteintech) antibodies, incubated overnight at 4°C. The α‐tubulin antibody is from Abcam (catalog number: ab4074; 1:10000 dilution) was used as an internal control.

### 4.21 Statistical Analysis

All quantitative results are expressed as the mean ± standard deviation. Comparisons for statistical analyses were generated by a blinded counter. Multiple comparisons were performed by one‐way analysis of variance (ANOVA) followed by post‐hoc tests and Student's t‐test accomplished comparison between two groups. Analyses were performed by using GraphPad Prism version 9.0 software. Statistical significance (two‐tailed) was identified as compared with a control group. *P* <0.05 (marked as *), *P* < 0.01 (marked as **), and *P* < 0.001 (marked as ***).

## Author Contributions

W.C. conceived and designed the project, conducted the experiments, analyzed the data, and wrote the original manuscript. H.J., Z.L., W.Z., N.Z., and Y.X. contributed to the data curation, investigation, and methodology. Y.H., J.N. and S.G. supervised the project and revised the manuscript. All authors have approved the final version of the manuscript.

## Conflicts of Interest

W. C. is an inventor on a patent application related to this work. The other authors declare no conflicts of interest.

## Supporting information




**Supporting File**: advs74946‐sup‐0001‐SuppMat.doc.

## Data Availability

The data that support the findings of this study are available from the corresponding author upon reasonable request.

## References

[1] J. S. Harrington , S. W. Ryter , M. Plataki , D. R. Price , and A. M. K. Choi , “Mitochondria in Health, Disease, and Aging,” Physiological Reviews 103, no. 4 (2023): 2349–2422, 10.1152/physrev.00058.2021. 37021870 PMC10393386

[2] T. Lima , T. Y. Li , A. Mottis , and J. Auwerx , “Pleiotropic Effects of Mitochondria in Aging,” Nature Aging 2, no. 3 (2022): 199–213, 10.1038/s43587-022-00191-2. 37118378

[3] A. A. Parkhitko , E. Filine , and M. Tatar , “Combinatorial Interventions in Aging,” Nature Aging 3, no. 10 (2023): 1187–1200, 10.1038/s43587-023-00489-9. 37783817 PMC11194689

[4] M. C. B. D'Alessandro , S. Kanaan , M. Geller , D. Praticò , and J. P. L. Daher , “Mitochondrial Dysfunction in Alzheimer's Disease,” Ageing Research Reviews 107 (2025): 102713, 10.1016/j.arr.2025.102713. 40023293

[5] J. A. Amorim , G. Coppotelli , A. P. Rolo , C. M. Palmeira , J. M. Ross , and D. A. Sinclair , “Mitochondrial and Metabolic Dysfunction in Ageing and Age‐Related Diseases,” Nature Reviews Endocrinology 18, no. 4 (2022): 243–258, 10.1038/s41574-021-00626-7. PMC9059418 35145250

[6] X. Qin , H. Li , H. Zhao , L. Fang , and X. Wang , “Enhancing Healthy Aging With Small Molecules: A Mitochondrial Perspective,” Medicinal Research Reviews 44, no. 4 (2024): 1904–1922, 10.1002/med.22034. 38483176

[7] A. Sharma , H. J. Smith , P. Yao , and W. B. Mair , “Causal Roles of Mitochondrial Dynamics in Longevity and Healthy Aging,” EMBO Reports 20, no. 12 (2019): 48395, 10.15252/embr.201948395. PMC6893295 31667999

[8] I. Vercellino and L. A. Sazanov , “The Assembly, Regulation and Function of the Mitochondrial Respiratory Chain,” Nature Reviews Molecular Cell Biology 23, no. 2 (2022): 141–161, 10.1038/s41580-021-00415-0. 34621061

[9] B. Kadenbach , “Regulation of Cytochrome *c* Oxidase Contributes to Health and Optimal Life,” World Journal of Biological Chemistry 11, no. 2 (2020): 52–61, 10.4331/wjbc.v11.i2.52. 33024517 PMC7520645

[10] W. Yin , R. Li , X. Feng , and Y. J. Kang , “The Involvement of Cytochrome *c* Oxidase in Mitochondrial Fusion in Primary Cultures of Neonatal Rat Cardiomyocytes,” Cardiovascular Toxicology 18, no. 4 (2018): 365–373, 10.1007/s12012-018-9447-1. 29396798

[11] K. Takahashi , I. Ohsawa , T. Shirasawa , and M. Takahashi , “Early‐onset Motor Impairment and Increased Accumulation of Phosphorylated α‐synuclein in the Motor Cortex of Normal Aging Mice Are Ameliorated by Coenzyme Q,” Experimental Gerontology 81 (2016): 65–75, 10.1016/j.exger.2017.09.002. 27143639

[12] K. Takahashi , I. Ohsawa , T. Shirasawa , and M. Takahashi , “Optic Atrophy 1 Mediates Coenzyme Q‐Responsive Regulation of Respiratory Complex IV Activity in Brain Mitochondria,” Experimental Gerontology 98 (2017): 217–223, 10.1016/j.exger.2017.09.002. 28890359

[13] S. Miwa , S. Kashyap , E. Chini , and T. von Zglinicki , “Mitochondrial Dysfunction in Cell Senescence and Aging,” The Journal of Clinical Investigation 132, no. 13 (2022), 10.1172/JCI158447. PMC9246372 35775483

[14] J. C. Ren , I. Rebrin , V. Klichko , W. C. Orr , and R. S. Sohal , “Cytochrome *c* Oxidase Loses Catalytic Activity and Structural Integrity During the Aging Process in Drosophila Melanogaster,” Biochemical and Biophysical Research Communications 401, no. 1 (2010): 64–68, 10.1016/j.bbrc.2010.09.009. 20833144 PMC2964050

[15] I. Soro‐Arnaiz , Q. O. Y. Li , M. Torres‐Capelli , et al., “Role of Mitochondrial Complex IV in Age‐Dependent Obesity,” Cell Reports 16, no. 11 (2016): 2991–3002, 10.1016/j.celrep.2016.08.041. 27626667

[16] G. Reichart , J. Mayer , C. Zehm , et al., “Mitochondrial Complex IV Mutation Increases Reactive Oxygen Species Production and Reduces Lifespan in Aged Mice,” Acta Physiologica 225, no. 4 (2019): 13214, 10.1111/apha.13214. 30376218

[17] F. M. Morais , A. M. Ribeiro , F. A. Moreira , and P. V. G. Silva , “Systematic Review and Meta‐analysis on the Role of Mitochondrial Cytochrome *c* Oxidase in Alzheimer's Disease,” Acta Neuropsychiatrica 33, no. 2 (2021): 55–64, 10.1017/neu.2020.43. 33256871

[18] Y. A. Hajam , R. Rani , S. Y. Ganie , et al., “Oxidative Stress in Human Pathology and Aging: Molecular Mechanisms and Perspectives,” Cells 11, no. 3 (2022): 552, 10.3390/cells11030552. 35159361 PMC8833991

[19] M. Tavallaie , R. Voshtani , X. Deng , et al., “Moderation of Mitochondrial Respiration Mitigates Metabolic Syndrome of Aging,” Proceedings of the National Academy of Sciences 117, no. 18 (2020): 9840–9850, 10.1073/pnas.1917948117. PMC7211973 32303655

[20] S. Campesan , I. del Popolo , K. Marcou , et al., “Bypassing Mitochondrial Defects Rescues Huntington's Phenotypes in Drosophila,” Neurobiology of Disease 185 (2023): 106236. 37495179 10.1016/j.nbd.2023.106236

[21] R. Yan , J. Ren , J. Wen , et al., “Enzyme Therapeutic for Ischemia and Reperfusion Injury in Organ Transplantation,” Advanced Materials 34 (2022): 2105670, 10.1002/adma.202105670. 34617335

[22] Y. Guo , T. Guan , K. Shafiq , et al., “Mitochondrial Dysfunction in Aging,” Ageing Research Reviews 88 (2023): 101955, 10.1016/j.arr.2023.101955. 37196864

[23] T. Liu , B. Lin , Y. Zhang , et al., “Modulating Lysine Crotonylation in Ulcerative Colitis Maintains Mitochondrial Homeostasis,” Exploration 5 (2025): 20240129, 10.1002/EXP.20240129. 41476658 PMC12752630

[24] Y. Tian , G. Garcia , Q. Bian , et al., “Mitochondrial Stress Induces Chromatin Reorganization to Promote Longevity and UPR^mt^ ,” Cell 165, no. 5 (2016): 1197–1208, 10.1016/j.cell.2016.04.011. 27133166 PMC4889216

[25] J. Yuan , S.‐Y. Chang , S.‐G. Yin , et al., “Two Conserved Epigenetic Regulators Prevent Healthy Ageing,” Nature 579, no. 7797 (2020): 118–122, 10.1038/s41586-020-2037-y. 32103178

[26] W. Cong , L. Meng , Y. Pan , et al., “Mitochondrial‐Mimicking Nanozyme‐Catalyzed Cascade Reactions for Aging Attenuation,” Nano Today 48 (2023): 101757, 10.1016/j.nantod.2023.101757.

[27] R. Zhang , B. Jiang , K. Fan , L. Gao , and X. Yan , “Designing Nanozymes for In *Vivo* Applications,” Nature Reviews Bioengineering 2, no. 10 (2024): 849–868, 10.1038/s44222-024-00205-1.

[28] W. Zuo , N. Liu , Z. Chang , et al., “Single‐Site Bimetallic Nanosheet for Imaging Guided Mutually‐Reinforced Photothermal‐Chemodynamic Therapy,” Chemical Engineering Journal 442 (2022): 136125, 10.1016/j.cej.2022.136125.

[29] Y. Shi , H. Li , D. Chu , et al., “Rescuing Nucleus Pulposus Cells From Senescence via Dual‐Functional Greigite Nanozyme to Alleviate Intervertebral Disc Degeneration,” Advanced Science 10, no. 25 (2023): 2300988, 10.1002/advs.202300988. 37400370 PMC10477883

[30] X. Du , Z. Dong , Y. Yan , et al., “Immunomodulatory Nanozymes Eradicate Intracellular Infections and Rescue Immunoparalysis for Treating Multidrug‐Resistant Bacterial Sepsis,” Exploration 5 (2025): 20250127, 10.1002/EXP.20250127. 41163807 PMC12561197

[31] L. Ma , Q. Zhang , J. Guo , et al., “Bimetallic Single‐Atom Nanozyme With Enhanced Multienzyme‐Mimicking Activity for Osteoarthritis Treatment via Reprogramming Inflammatory and Metabolic Microenvironment,” ACS Applied Materials & Interfaces 17 (2025): 66391–66406, 10.1021/acsami.5c17013. 41292118

[32] N. Singh and G. Mugesh , “CeVO_4_ Nanozymes Catalyze the Reduction of Dioxygen to Water Without Releasing Partially Reduced Oxygen Species,” Angewandte Chemie International Edition 58, no. 23 (2019): 7797–7801, 10.1002/anie.201903427. 30950157

[33] X. Huang , S. Tang , X. Mu , et al., “Freestanding Palladium Nanosheets With Plasmonic and Catalytic Properties,” Nature Nanotechnology 6, no. 1 (2011): 28–32, 10.1002/adma.202203236. 21131956

[34] G. Fang , W. Li , X. Shen , et al., “Differential Pd‐nanocrystal Facets Demonstrate Distinct Antibacterial Activity against Gram‐Positive and Gram‐Negative Bacteria,” Nature Communications 9, no. 1 (2018): 129, 10.1038/s41467-017-02502-3. PMC5760645 29317632

[35] X. Yang , X. Cao , X. Wang , et al., “Palladium Nanoparticles Degrade Advanced Glycation End Products via Valosin‐Containing Protein Mediated Autophagy to Attenuate High‐Glucose/High‐Fat‐Induced Intervertebral Disc Degeneration,” Exploration 5 (2025): 20230174, 10.1002/EXP.20230174. 40395754 PMC12087406

[36] S. Li , B. Xu , M. Lu , et al., “Tensile‐Strained Palladium Nanosheets for Synthetic Catalytic Therapy and Phototherapy,” Advanced Materials 34, no. 32 (2022): 2202609, 10.1002/adma.202202609. 35610760

[37] T. Kaletta and M. O. Hengartner , “Finding Function in Novel Targets: *C. elegans* as a Model Organism,” Nature Reviews Drug Discovery 5, no. 5 (2006): 387–399, 10.1038/nrd2031. 16672925

[38] P. V. S. Vasileiou , K. Evangelou , K. Vlasis , et al., “Mitochondrial Homeostasis and Cellular Senescence,” Cells 8, no. 7 (2019): 686, 10.3390/cells8070686. 31284597 PMC6678662

[39] K. R. Gentry , L. M. Steele , M. M. Sedensky , and P. G. Morgan , “Early Developmental Exposure to Volatile Anesthetics Causes Behavioral Defects in *Caenorhabditis elegans* ,” Anesthesia & Analgesia 116, no. 1 (2013): 185–189, 10.1213/ANE.0. 23223110 PMC3607665

[40] M. Chang , Z. Hou , M. Wang , et al., “Single‐Atom Pd Nanozyme for Ferroptosis‐Boosted Mild‐Temperature Photothermal Therapy,” Angewandte Chemie International Edition 60, no. 23 (2021): 12971–12979, 10.1002/anie.202101924. 33772996

[41] R. Narayanan and M. A. El‐Sayed , “Effect of Catalysis on the Stability of Metallic Nanoparticles: Suzuki Reaction Catalyzed by PVP‐Palladium Nanoparticles,” Journal of the American Chemical Society 125, no. 27 (2003): 8340–8347, 10.1021/ja035044x. 12837106

[42] M. Chen , Z. Wang , J. Shu , et al., “Mimicking a Natural Enzyme System: Cytochrome *c* Oxidase‐Like Activity of Cu_2_O Nanoparticles by Receiving Electrons From Cytochrome *c* ,” Inorganic Chemistry 56, no. 16 (2017): 9400–9403, 10.1021/acs.inorgchem.7b01393. 28753305

[43] X. Ma , L.‐H. Zhang , L.‐R. Wang , et al., “Single‐Walled Carbon Nanotubes Alter Cytochrome *c* Electron Transfer and Modulate Mitochondrial Function,” ACS Nano 6, no. 12 (2012): 10486–10496, 10.1021/nn302457v. 23171082 PMC3548237

[44] W. Cong , Y. Wang , C. Yuan , et al., “Dietary Cobalt Oxide Nanoparticles Alleviate Aging Through Activation of Mitochondrial UPR in *Caenorhabditis elegans* ,” Theranostics 13, no. 10 (2023): 3276–3289, 10.7150/thno.81817. 37351160 PMC10283066

[45] W. Cong , R. Bai , Y. Li , L. Wang , and C. Chen , “Selenium Nanoparticles as an Efficient Nanomedicine for the Therapy of Huntington's Disease,” ACS Applied Materials & Interfaces 11, no. 38 (2019): 34725–34735, 10.1021/acsami.9b12319. 31479233

