# Supplementary material for: Mitochondrial Enzymes Mimetic Ultrasmall Palladium Nanozymes Prevent Senescence and Neurodegeneration Through Metabolic Reprogramming
Source: Adv Sci (Weinh). 2026 Apr 14;13(32):e23931. doi: 10.1002/advs.202523931 (PMC13252661; doi:10.1002/advs.202523931)
Supplement: Supplementary file 1 — Supporting File: advs74946‐sup‐0001‐SuppMat.doc. [file ADVS-13-e23931-s001.doc]

**Supporting Information**

[**Mitochondrial enzymes mimetic ultrasmall**](https://www.sciencedirect.com/science/article/pii/S0142961221000570) **palladium nan****ozymes prevent** **senescence and neurodegeneration** **through metabolic reprogramming**

Wenshu Cong, Haiming Jing, Zinan Li, Wenjing Zhang, Nan Zhang, Shan Gao*, Yuanyu Huang*, Junyu Ning*

W. Cong, H. Jing, Z. Li, W. Zhang, N. Zhang, S. Gao, J. Ning

Beijing Center for Disease Prevention and Control, Beijing Key Laboratory of Diagnostic and Traceability Technologies for Food Poisoning, Beijing 100013, China

Email: [njy_med@hotmail.com](mailto:njy_med@hotmail.com); [gaoshan20250110@163.com](mailto:gaoshan20250110@163.com)

W. Cong, Y. Huang

School of Life Science; School of Interdisciplinary Science; Key Laboratory of Molecular Medicine and Biotherapy; Key Laboratory of Medical Molecule Science and Pharmaceutics Engineering, Beijing Institute of Technology, Beijing 100081, China

Email: [yyhuang@bit.edu.cn](mailto:yyhuang@bit.edu.cn)

W. Cong, H. Jing, Z. Li, J. Ning

School of Public Health, Capital Medical University, Beijing 10081, China

Y. Xie

Department of Biology, School of Sciences and Humanities, Nazarbayev University, Astana 010000, Kazakhstan.

Y. Huang

School of Medical Engineering; School of Interdisciplinary Science; Affiliated Zhuhai People's Hospital, Beijing Institute of Technology, Zhuhai 519088, China.


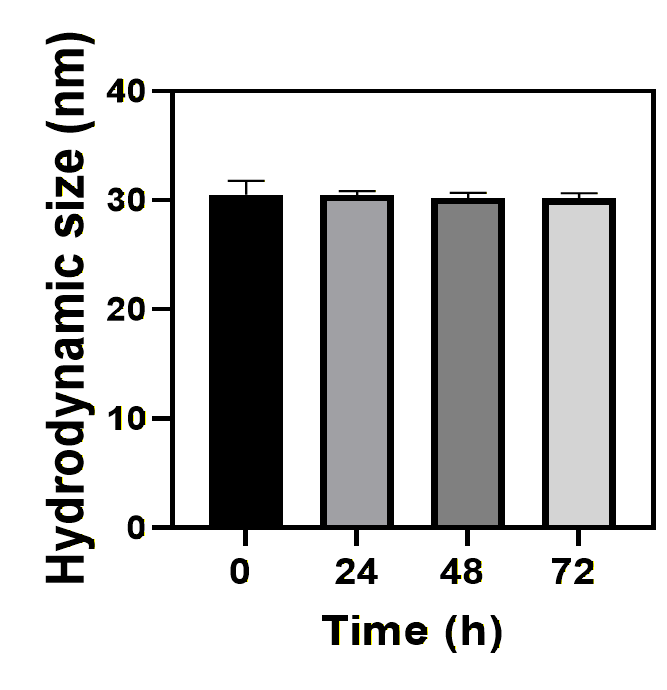


**Figure S1.** Changes in the hydrodynamic size of PdP NPs in solution measured by dynamic light scattering (DLS). Data represent means value ± standard errors. n = 3; *P*_24_ > 0.9999, *P*_48_ = 0.9744, *P*_72_ = 0.9481. Two-sided one-way ANOVA test followed by a Tukey post hoc analysis.


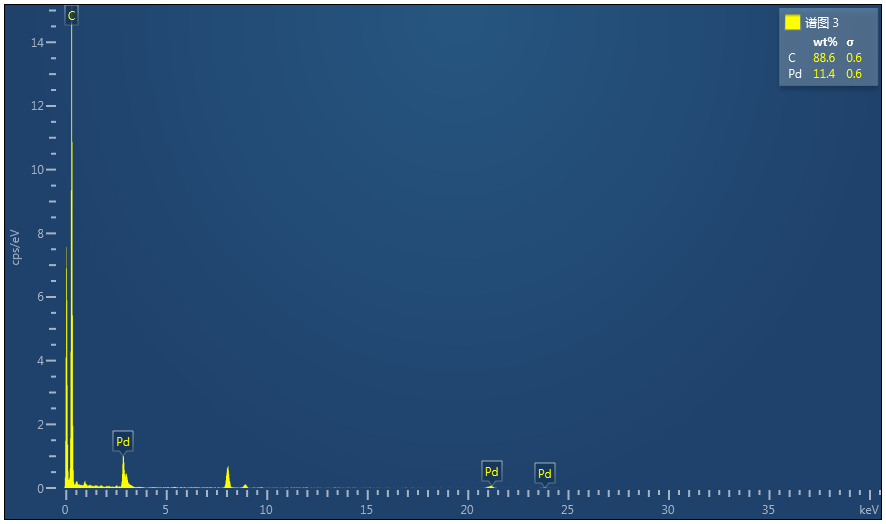


**Figure S2.** EDX analysis of PdP NPs.


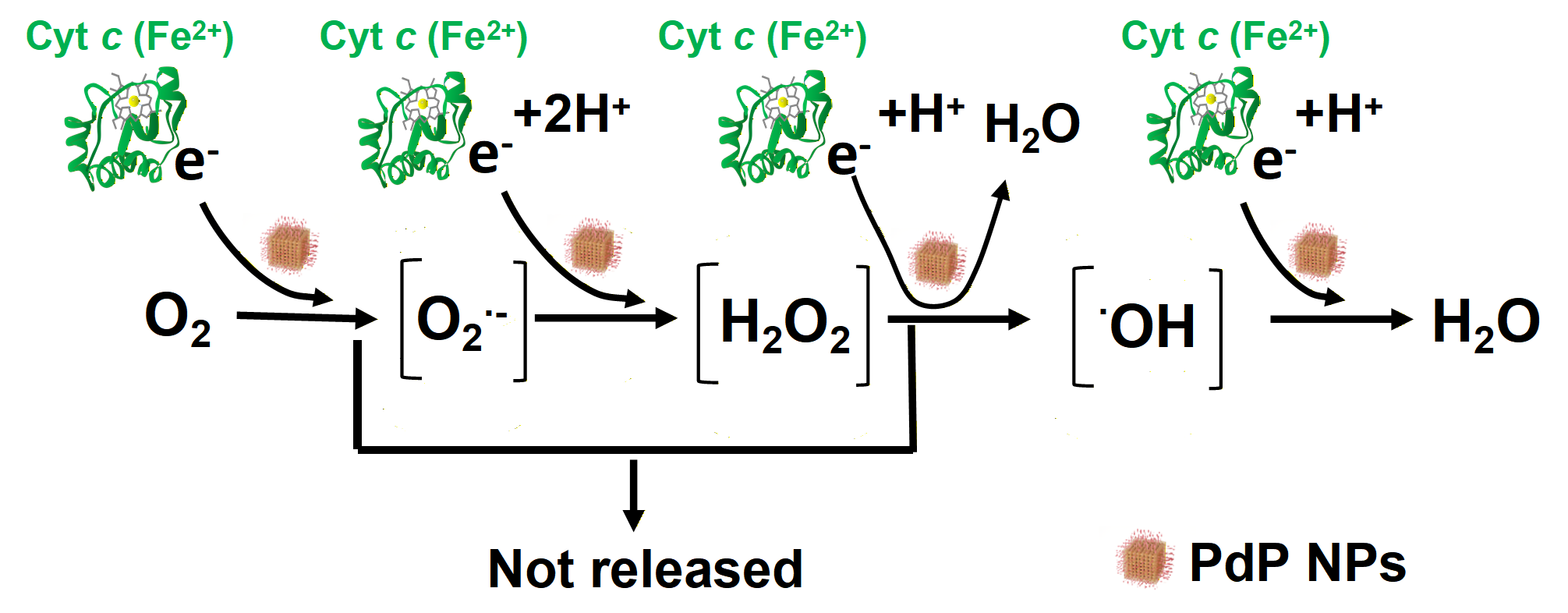


**Figure S3.** Scheme showing four electron reduction of O_2_ to water and the intermediates of the reaction.


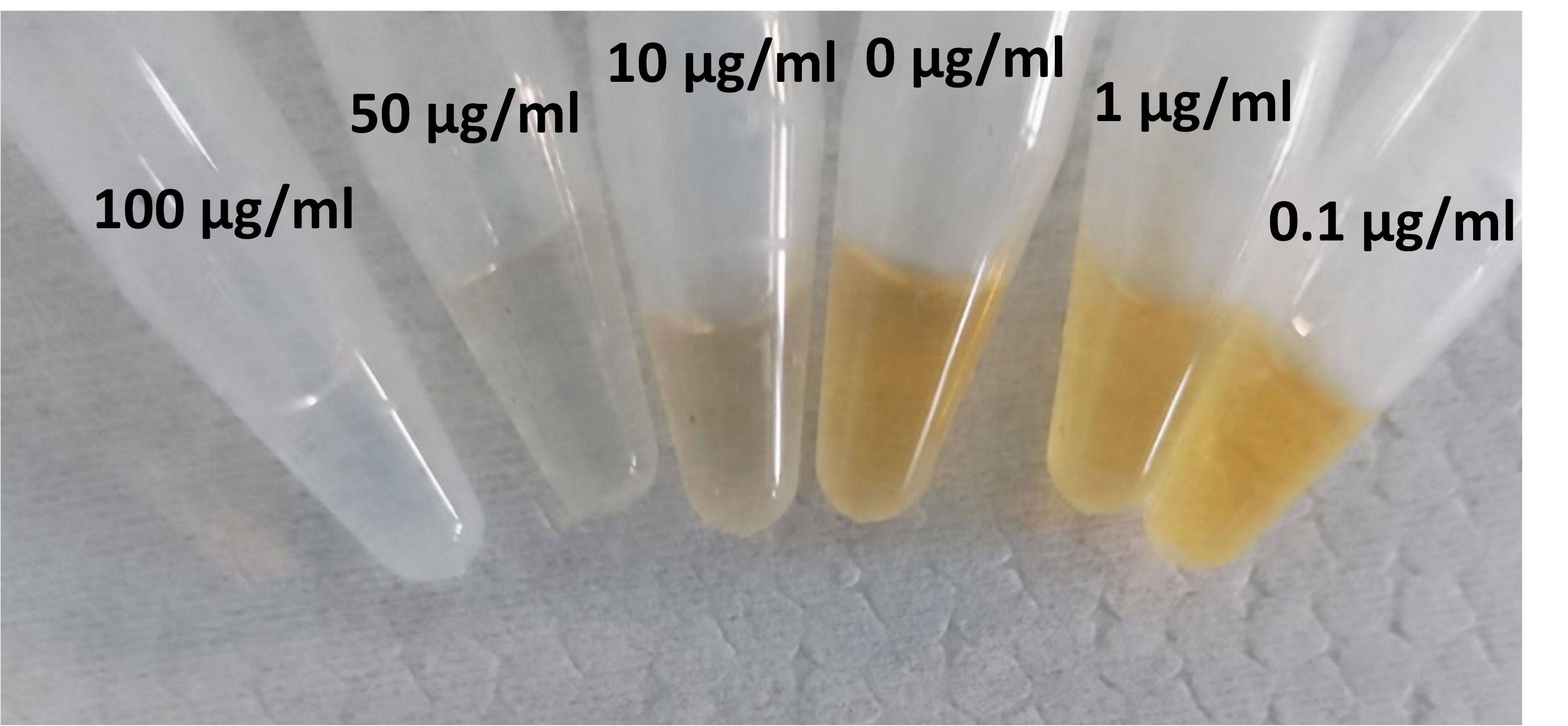


**Figure S4.** The change of color of WST-8 when added with different concentrations (0-100 μg ml^-1^) of PdP NPs, indicating the reduced production of water-soluble formation.

**
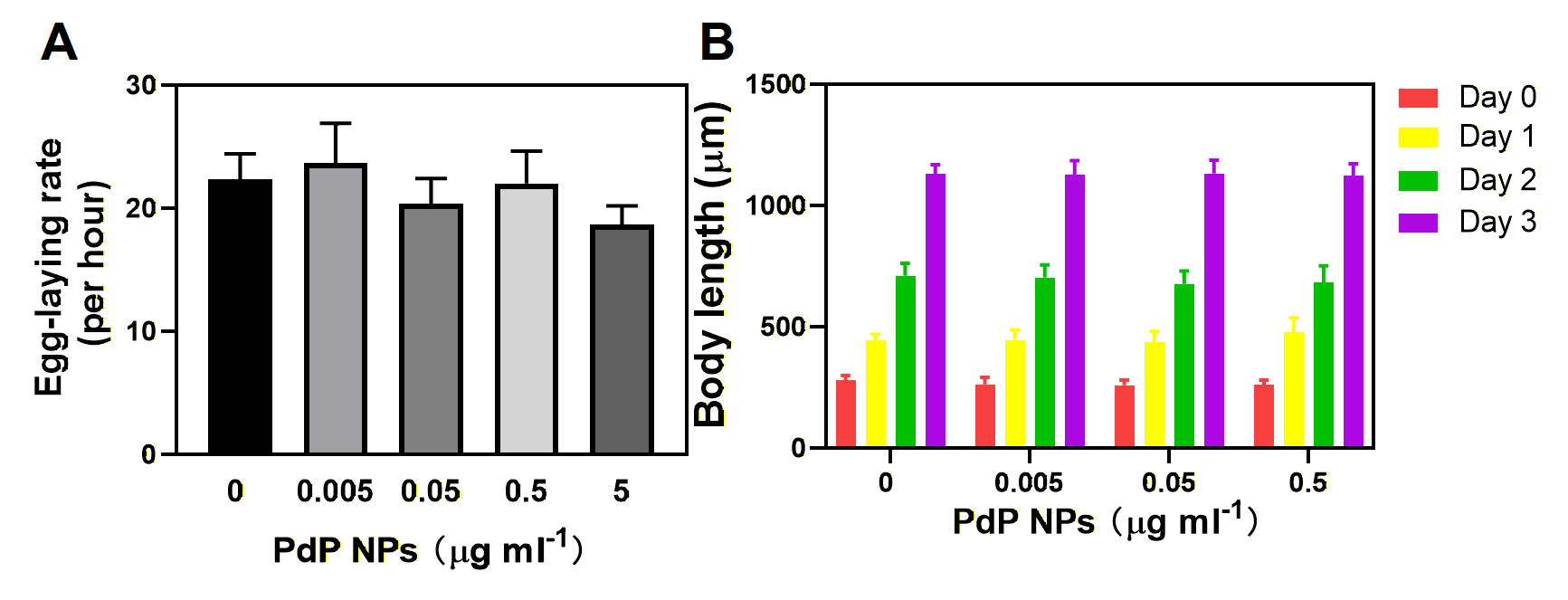
**

**Figure S5.** Biosafety assessment of PdP NPs on *C. elegans*. (A) Effects of PdP NPs on fertility were shown by egg-laying rate of *C. elegans* (n = 3; *P*_0.005_ = 0.8949, *P*_0.05_ = 0.7004, *P*_0.5_ = 0.9991, *P*_5_ = 0.2432). (B) Body length of offspring from worms treated with PdP NPs (Day 0: n_0_ = 48, n_0.005_ = 22, n_0.05_ = 22, n_0.5_ = 23; Day 1: n_0_ = 22, n_0.005_ = 28, n_0.05_ = 20, n_0.5_ = 19; Day 2: n_0_ = 20, n_0.005_ = 32, n_0.05_ = 30, n_0.5_ = 25; Day 3: n_0_ = 21, n_0.005_ = 17, n_0.05_ = 22, n_0.5_ = 24). Wild-type worms at L1 larva stage were treated with PdP NPs with gradient doses of PdP NPs for three days. Bars represent means ± SD.

**Figure S6.** Comparison of ATP levels in *C. elegans* treated with PdP NPs or not on day 1 of adulthood (n = 3, *P* < 0.0001). The data was normalized by defining the mean value of control group as 100 %. ***P < 0.001. Two-sided Student’s t-test.

**Figure S7.** Comparison of ATP levels in mammalian HEK293T cells treated with PdP NPs or not (n = 3, *P* = 0.0032). The data was normalized by defining the mean value of control group as 100 %. **P < 0.05. Two-sided Student’s t-test


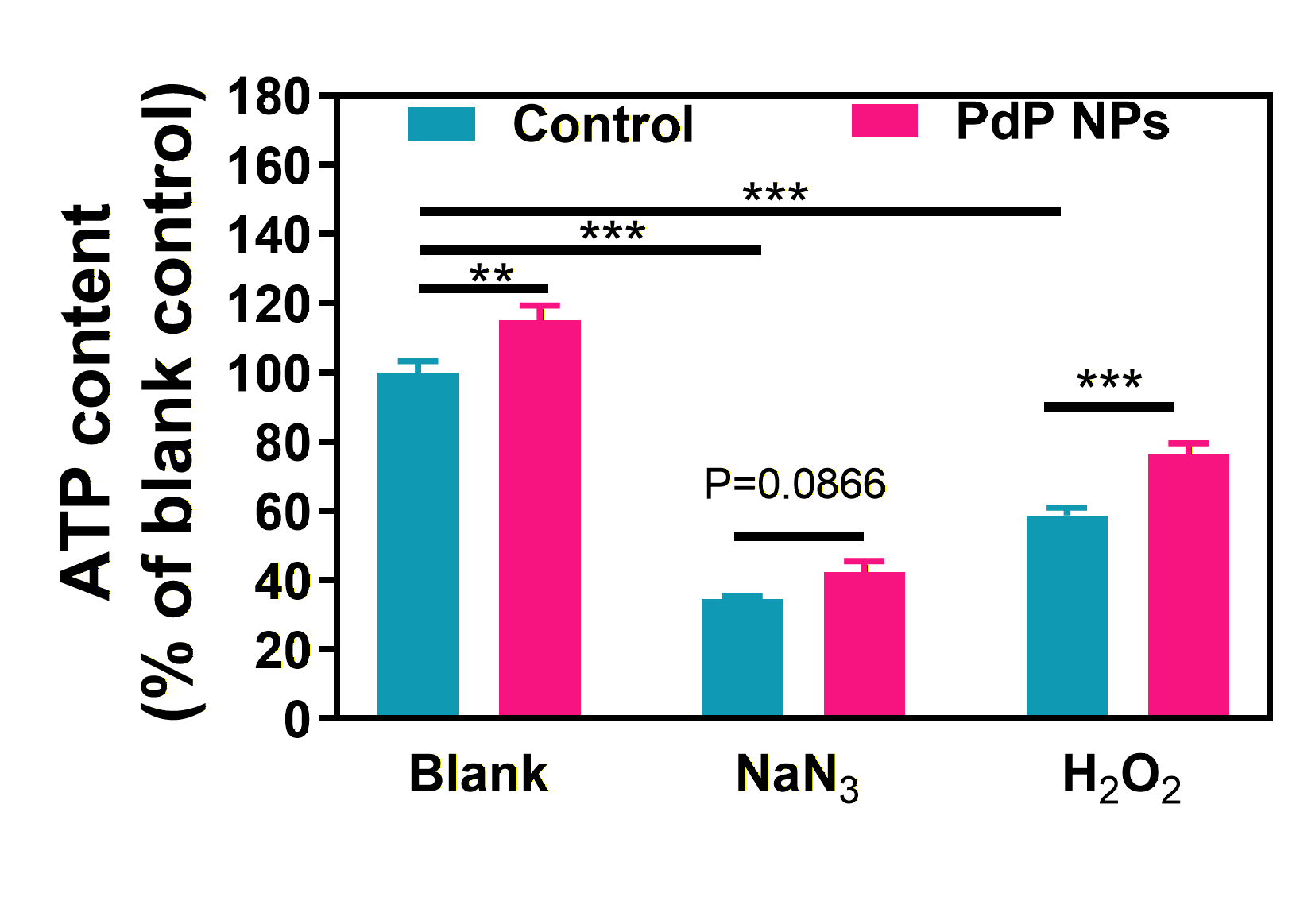


**Figure S8.** Evaluation of the effect of PdP NPs on ATP levels in NaN_3_ treated, H_2_O_2_ treated and blank treated mammalian SH-SY5Y cells (n = 3). The data was normalized by defining the mean value of blank control group as 100 % and set 0 as 0%. *P* = 0.0021 (PdP NPs versus blank control); *P* < 0.0001 (NaN_3_-only control versus blank control); *P* = 0.0866 (NaN_3_-treated PdP NPs group versus NaN_3_-only control); *P* < 0.0001 (H_2_O_2_-only control versus blank control); *P* = 0.0002 (H_2_O_2_-treated PdP NPs group versus H_2_O_2_-only control). *P < 0.05, **P < 0.01, ***P < 0.001. Two-sided one-way ANOVA test followed by a Tukey post hoc analysis.

**Figure S9.** The mRNA level of key metabolic genes *cts-1* (TCA cycle), *cox-4* (ETC chain enzyme complex IV), *hxk-1* (glycolysis) and *pyc-1*(gluconeogenesis) in worms on day 7 of adulthood (n = 3; *P_cts-1_* = 0.0177, *P_cox-4_* < 0.0001, *P_hxk-1_* = 0.1855, *P_pyc-1_* < 0.0001). The data was normalized by defining the mean value of control group as 1. *P < 0.05, ***P < 0.001. Two-sided one-way ANOVA test followed by the Sidak multiple comparisons test.


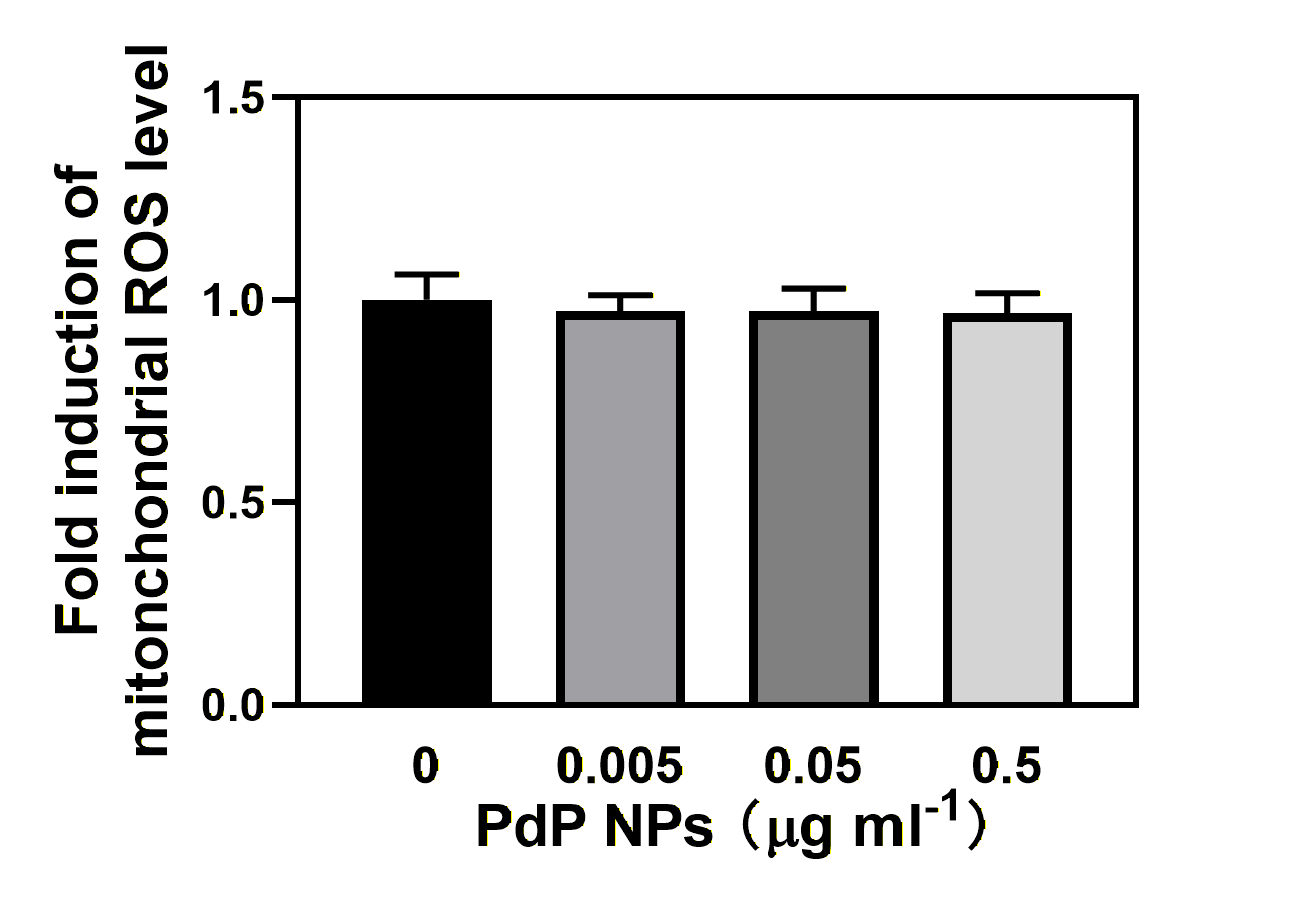


**Figure S10.** Quantified mitochondrial ROS of PdP NPs or mock treated SH-SY5Y cells (n = 6; *P*_0.005_ = 0.7891, *P*_0.05_ = 0.7916, *P*_0.5_ = 0.716). The data was normalized by defining the mean value of control group as 1. Two-sided one-way ANOVA test followed by a Tukey post hoc analysis.


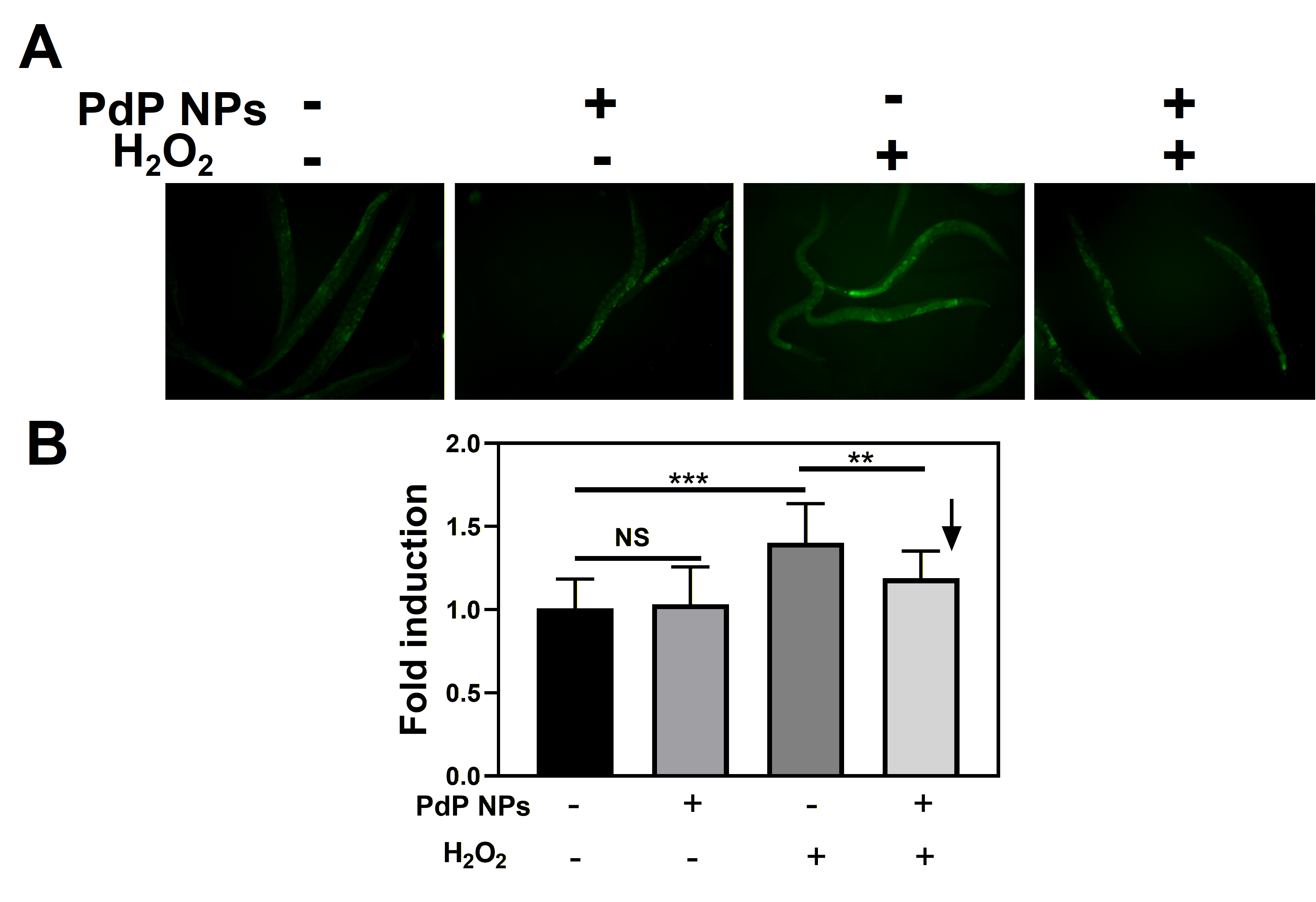


**Figure S11.** Representative pictures (A) and quantification (B) of whole-cell ROS in PdP NPs or mock-pretreated worms with or without H_2_O_2_ treatment (n_control_ = 32, n _PdP NPs_= 10, n_H2O2_ = 22, n_H2O2+PdP NPs_ = 27). *P* = 0.9843 (PdP NPs group versus mock control), *P* < 0.0001 (H_2_O_2_-only control versus mock control), *P* = 0.0012 (H_2_O_2_-treated PdP NPs group versus H_2_O_2_-only control). The data was normalized by defining the mean value of control group as 1. NS means no significance. **P < 0.01, ***P < 0.001. Two-sided one-way ANOVA test followed by a Tukey post hoc analysis.


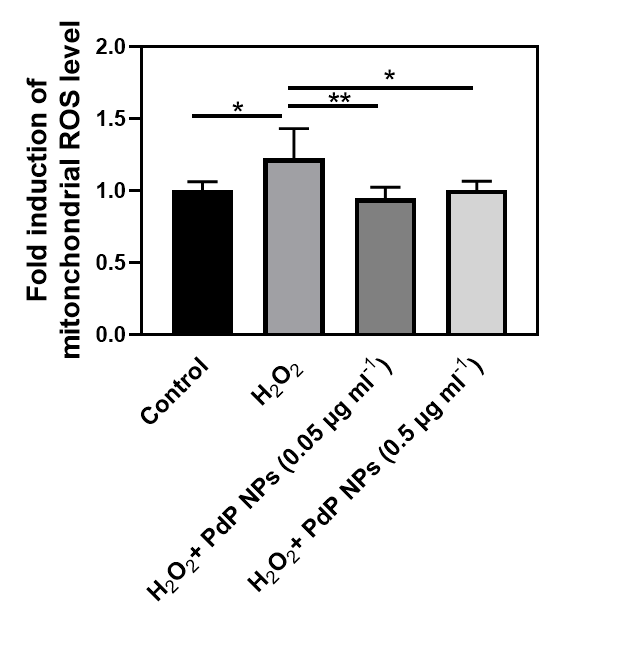


**Figure S12.** Quantification of MitoSOX fluorescence intensity of PdP NPs pretreated SH-SY5Y cells after H_2_O_2_ exposure for 1.5 h (n = 5-6). *P* = 0.0211 (H_2_O_2_-only control versus mock control), P = 0.0057 (H_2_O_2_-treated 0.05 μg ml^-1^ PdP NPs group versus H_2_O_2_-only control ), *P* = 0.0242 (H_2_O_2_-treated 0.5 μg ml^-1^ PdP NPs group versus H_2_O_2_-only control). The data was normalized by defining the mean value of control group as 1. *P < 0.05, **P < 0.01. Two-sided one-way ANOVA test followed by a Tukey post hoc analysis.

**
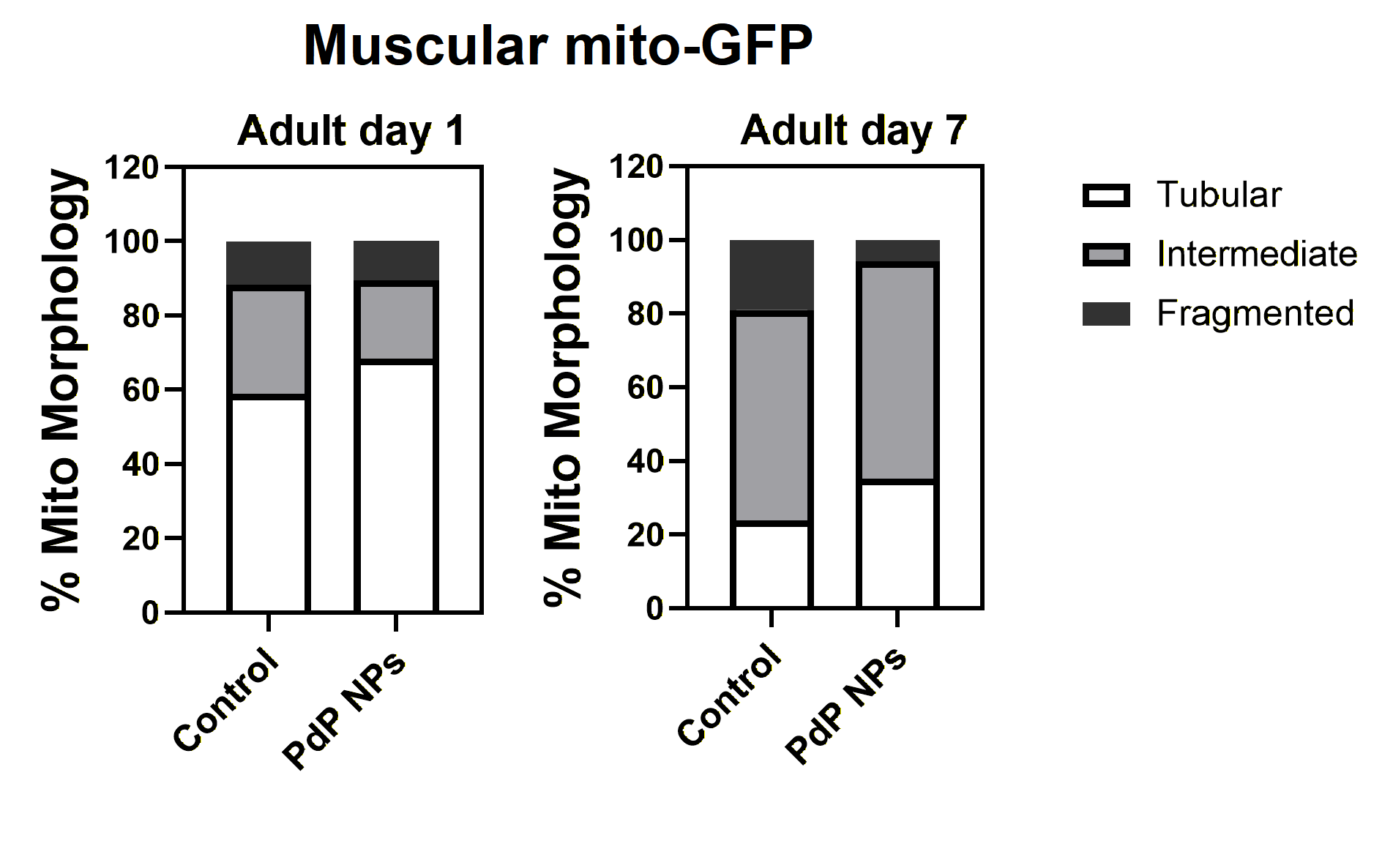
**

**Figure S13.** Quantification of the worms on adult day 1 and 7 with different mitochondria morphology (fragmented, intermediate or elongated) is shown (left, n_control_ = 17, n_PdP NPs_ = 19; Right, n_control_ = 21, n_PdP NPs_ = 17).

**Figure S14.** RT-qPCR analysis of mtDNA/nDNA ratio (mtce.26/ act-3) in PdP NPs or mock-pretreated wild-type worms on day 1 and day 7 of adulthood (n=3; left, *P* < 0.0001; Right, *P =* 0.0002). The data was normalized by defining the mean value of control group as 1. ***P < 0.001. Two-sided one-way ANOVA test followed by the Sidak multiple comparisons test.

.

**Figure S15.** Quantitation of nuclear genes encoding mitochondrial proteins (*F58F12.1, suclg-1, mrpl-47*) by qRT-PCR in N_2_ animals (n=3; *P_F58F12.1_ >* 0.9999, *P_suclg-1_ >* 0.9999, *P_mrpl-47_ =* 0.9945). The data was normalized by defining the mean value of control group as 1. Two-sided one-way ANOVA test followed by the Sidak multiple comparisons test.


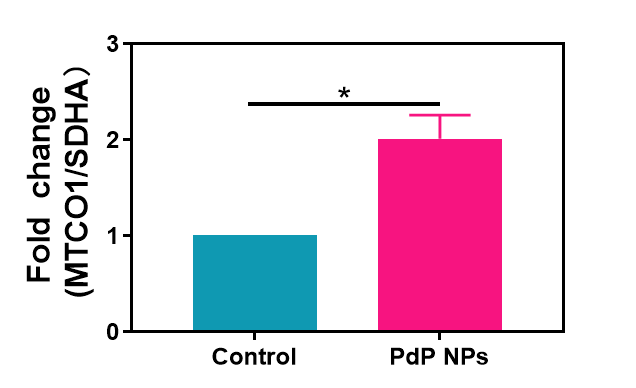


**Figure S16.** Quantitative analysis on the ratio of mtDNA-encoded MTCO1 and nDNA-encoded SDHA in *C. elegans* treated with PdP NPs (*P* = 0.0277). The data was normalized by defining the mean value of control group as 1. *P < 0.05. Two-sided Student’s t-test.


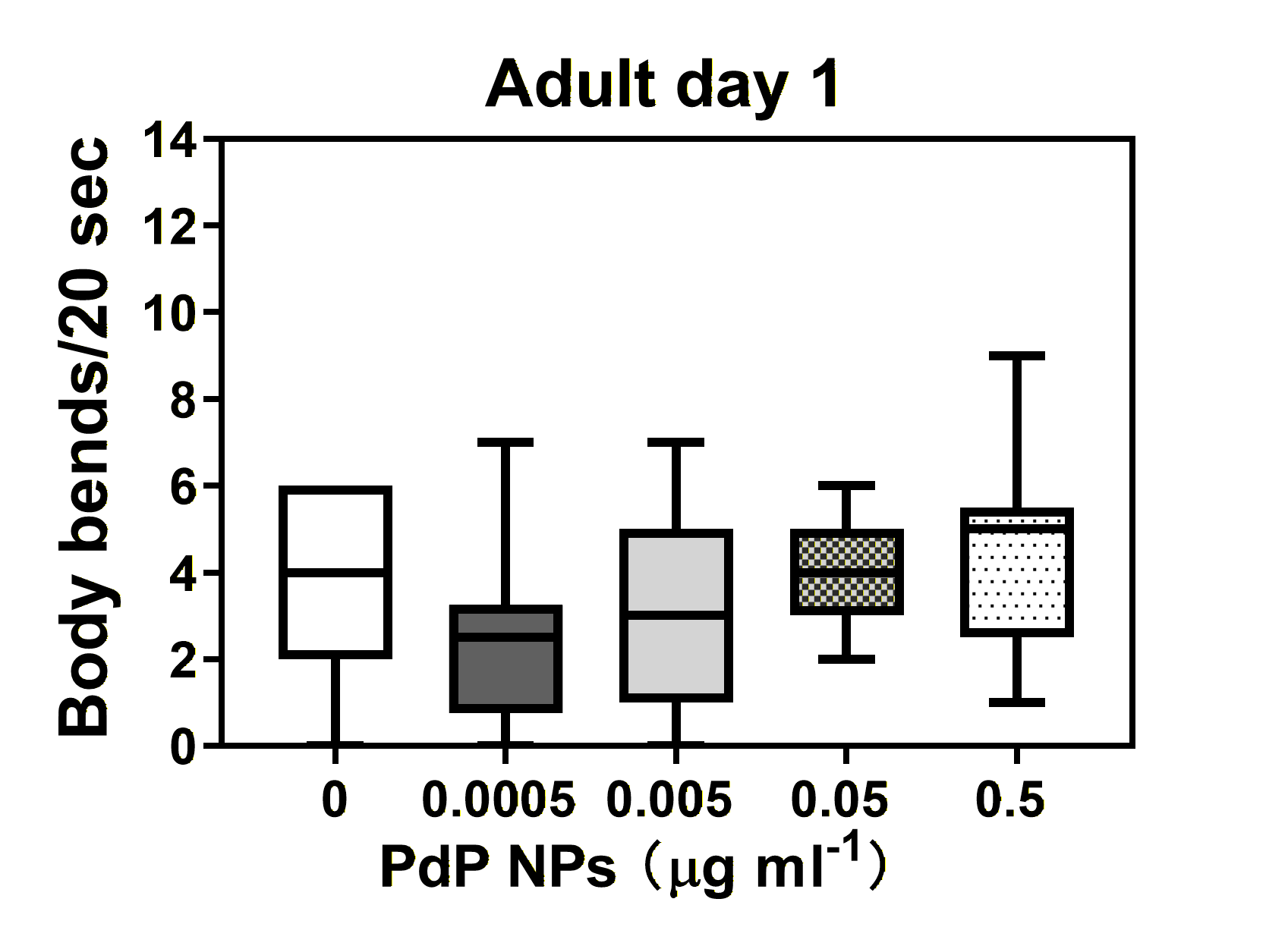


**Figure S17.** Body bends of young worms (adult day 1) pretreated with gradient concentrations of PdP NPs (n= 10-15; *P*_0.0005_ = 0.8621, *P*_0.005_ = 0.9931, *P*_0.05_ = 0.9561, *P*_0.5_ = 0.6337). Data represent means value ± standard errors. Two-sided one-way ANOVA test followed by a Tukey post hoc analysis.


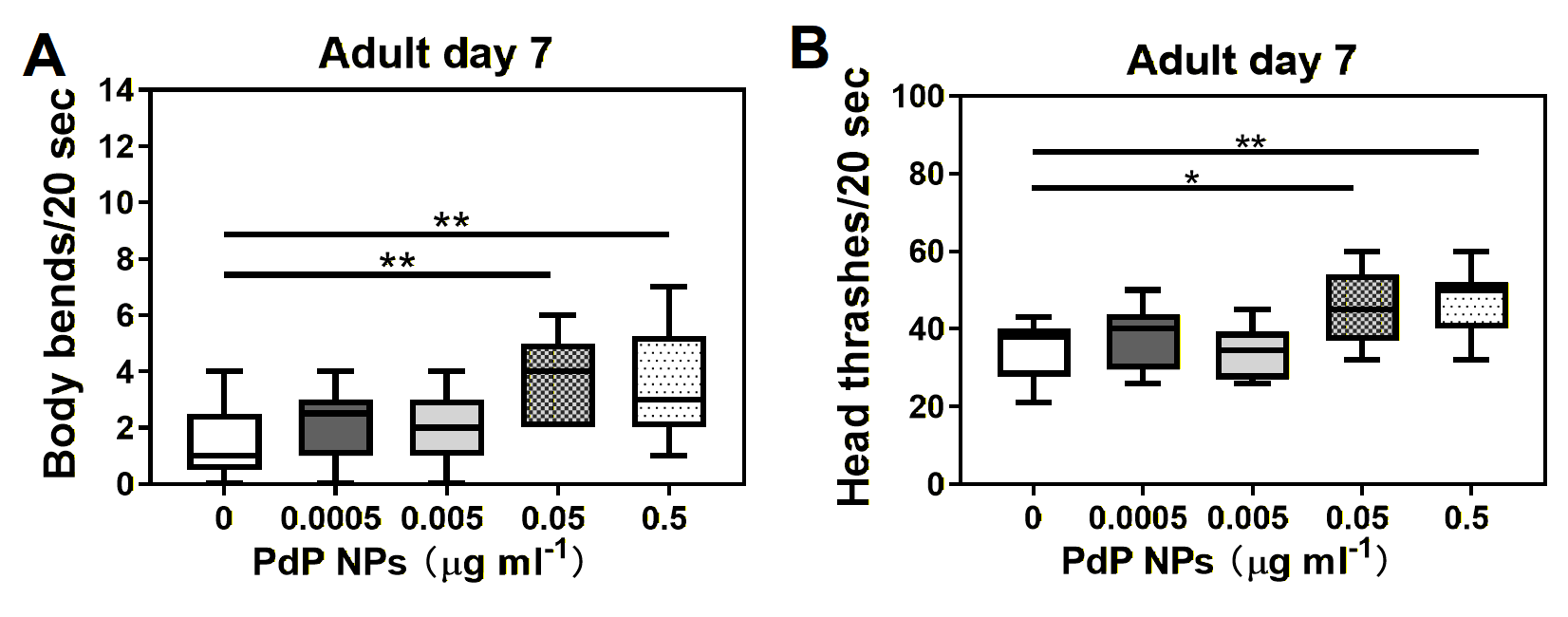


**Figure S18.** (A) Body bends of elderly worms (adult day 7) pretreated with diﬀerent concentrations of PdP NPs (n = 10-17, *P*_0.0005_ = 0.7879, *P*_0.005_ = 0.9166, *P*_0.05_ = 0.0019, *P*_0.5_ = 0.0028). (B) Head thrashes of elderly worms (adult day 7) pretreated with diﬀerent concentrations of PdP NPs (n= 10-12; *P*_0.0005_ = 0.8395, *P*_0.005_ > 0.9999, *P*_0.05_ = 0.0248, *P*_0.5_ = 0.0027). Data represent means value ± standard errors. *P < 0.05, **P < 0.01. Two-sided one-way ANOVA test followed by a Tukey post hoc analysis.


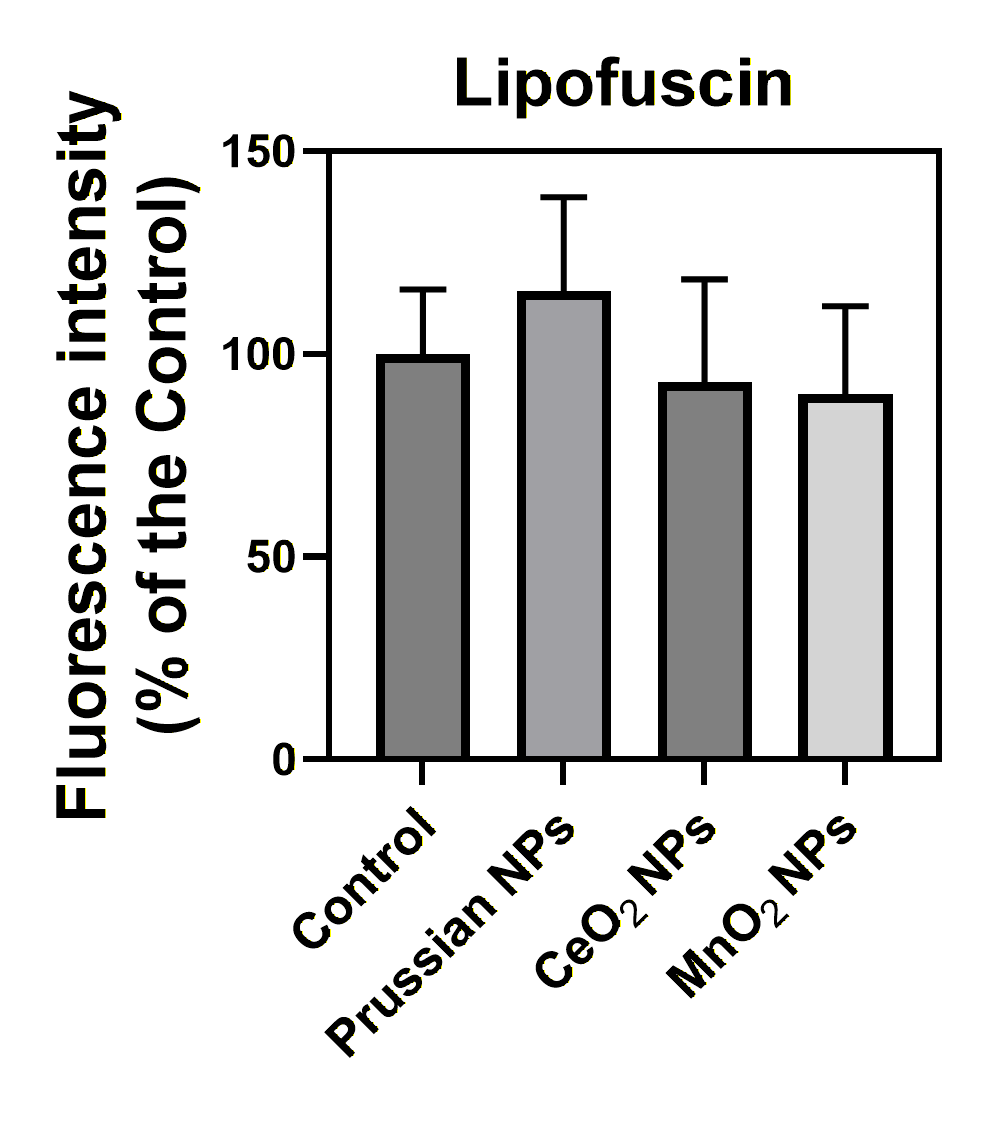


**Figure S19.** Quantification of lipofuscin fluorescence in nematodes pretreated with Prussian Blue NPs, CeO_2_ NPs, and MnO_2_ NPs by fluorescence microscope. Control, n = 16; Prussian NPs, n = 27, *P* = 0.1333; CeO_2_ NPs, n = 30, *P* = 0.753; MnO_2_ NPs, n = 21, *P* = 0.5496. The data was normalized by defining the mean value of control group as 100% and set 0 as 0%. Two-sided one-way ANOVA test followed by a Tukey post hoc analysis.

.


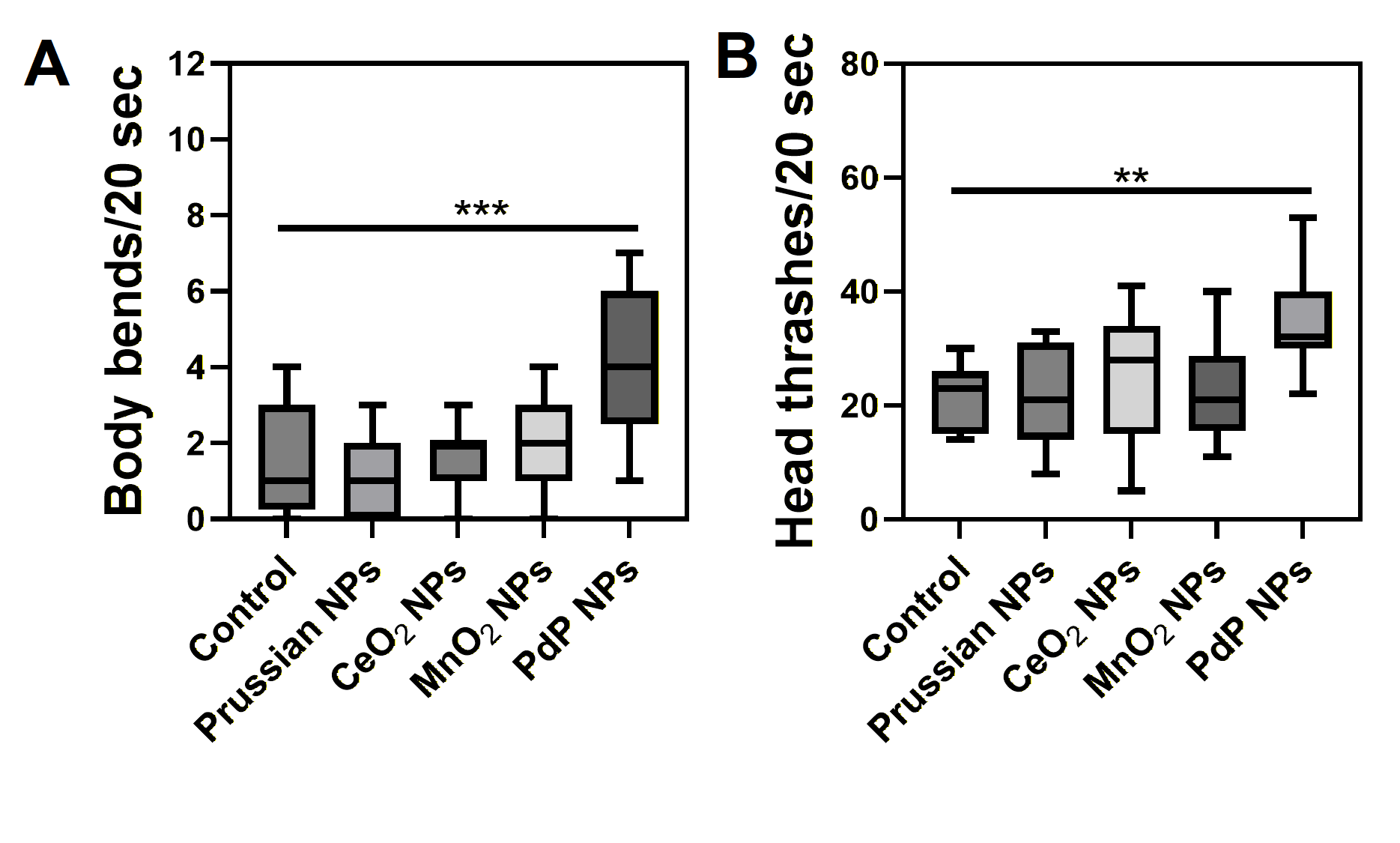


**Figure S20.** Effects of Prussian Blue NPs, CeO₂ NPs, MnO₂ NPs, and PdP NPs on the locomotor ability of *C. elegans*. (A) Body bends of day-7 adult worms pretreated with the indicated nanomaterials (Control, n = 20; Prussian NPs, n = 12, P = 0.941; CeO_2_ NPs, n = 16, P = 0.9983; MnO_2_ NPs, n = 15, P = 0.6432; PdP NPs, n = 17, *P* < 0.0001). (B) Head thrashes of day-7 adult worms pretreated with the indicated nanomaterials (Control, n = 15; Prussian NPs, n = 11, P > 0.9999; CeO_2_ NPs, n = 11, P = 0.7779; MnO_2_ NPs, n = 12, P = 0.9991; PdP NPs, n = 15, P = 0.0014). OP50 bacteria were heat-killed before being supplied as food. Data represent means value ± standard errors. **P < 0.01，***P < 0.001. Two-sided one-way ANOVA test followed by a Tukey post hoc analysis.


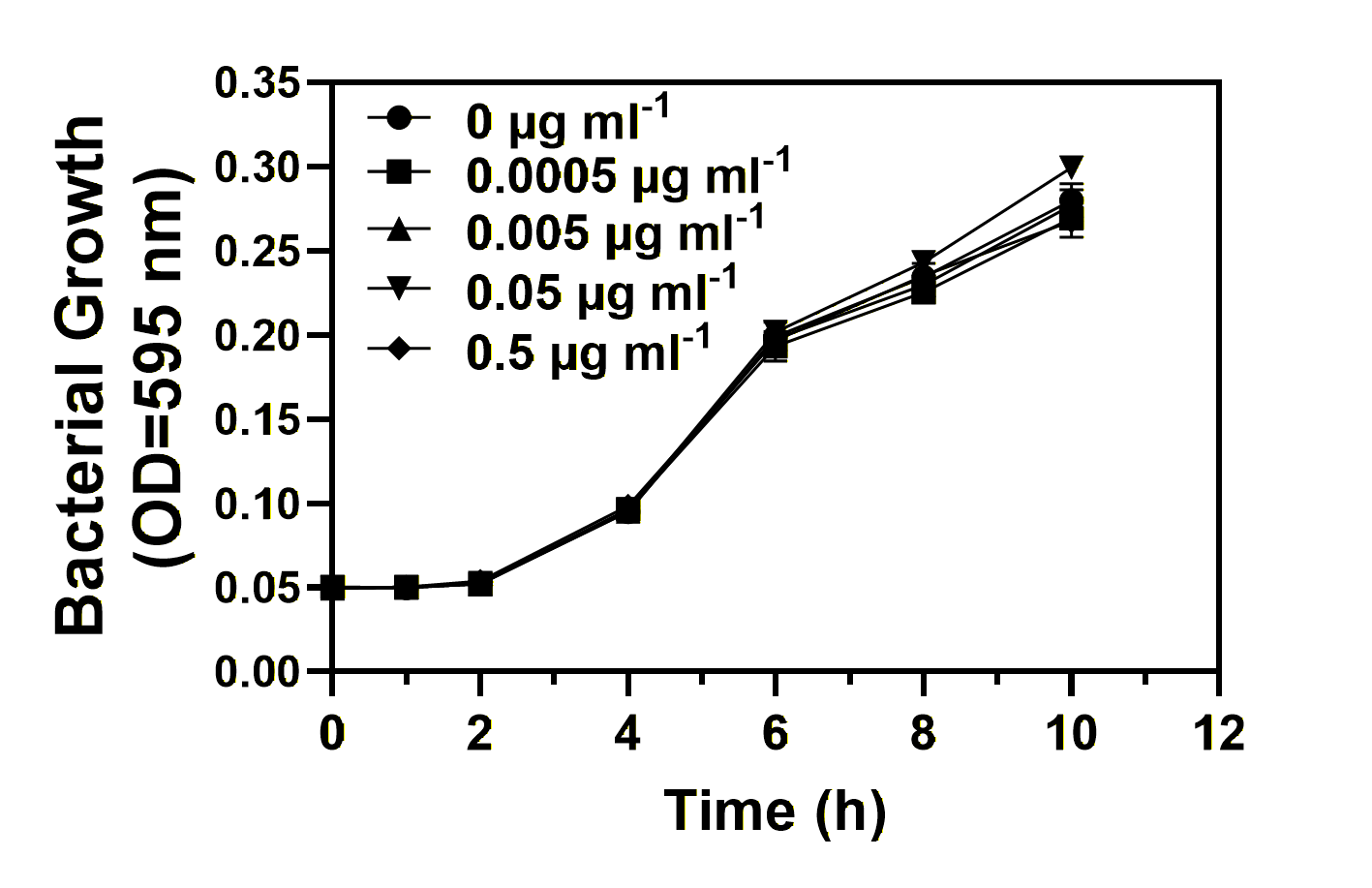


**Figure S21.** Escherichia coli strain OP50 cultured in liquid LB medium exhibited no growth inhibition upon exposure to gradient doses of PdP NPs (n=3; *P*_0.0005_ > 0.9999, *P*_0.005_ > 0.9999, *P*_0.05_ > 0.9999, *P*_0.5_ > 0.9999). Data represent means value ± standard errors. Two-sided one-way ANOVA test followed by a Tukey post hoc analysis.


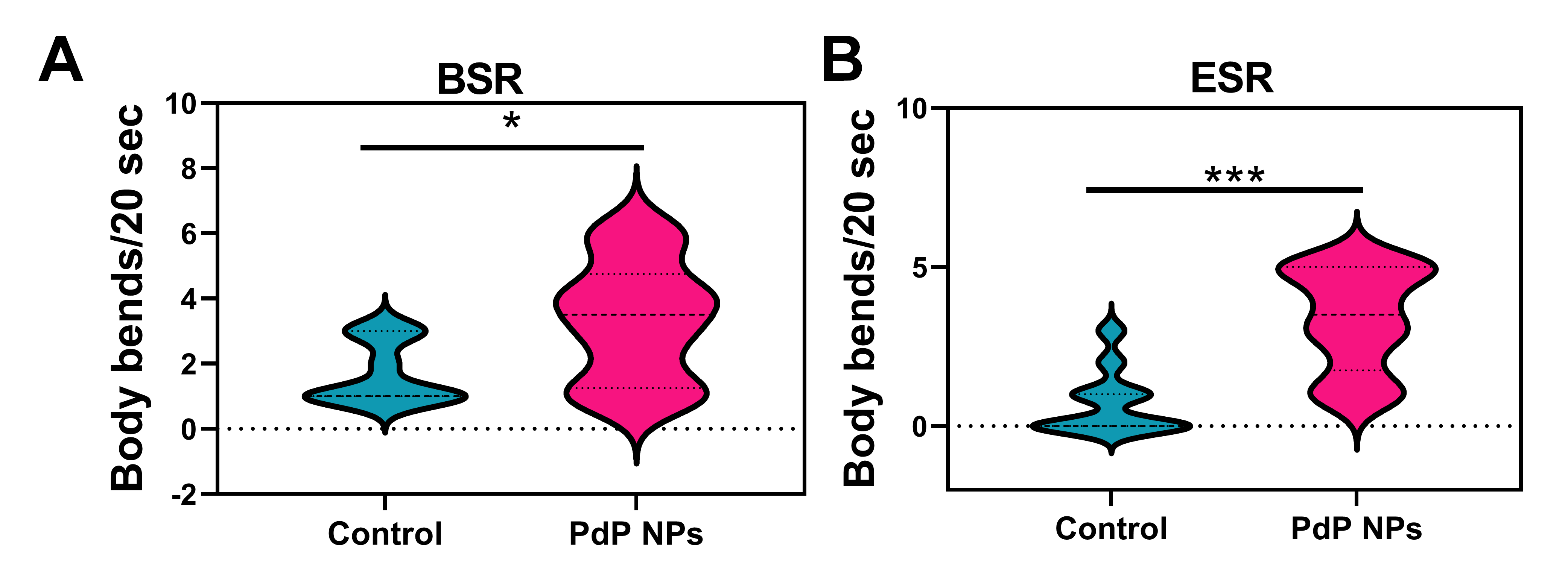


**Figure S22.** (A) Quantified basal slowing response (BSR) and (B) enhanced slowing response (ESR) indicated by body bends of worms on day 1 of adulthood (Left: n_control_ = 10, n_PdP_ = 12, *P* = 0.0191; Right: n_control_ = 11, n_PdP_ = 14, *P* < 0.0001). Data represent means value ± standard errors. *P < 0.05, ***P < 0.001. Two-sided Student’s t-test.

.

**Figure S23.** Lifespan curves of *dve-1* (fx0259) mutant worms in the presence of PdP NPs compared to untreated group (n = 3, *P* = 0.0128). *P < 0.05. Two-sided Student’s t-test.


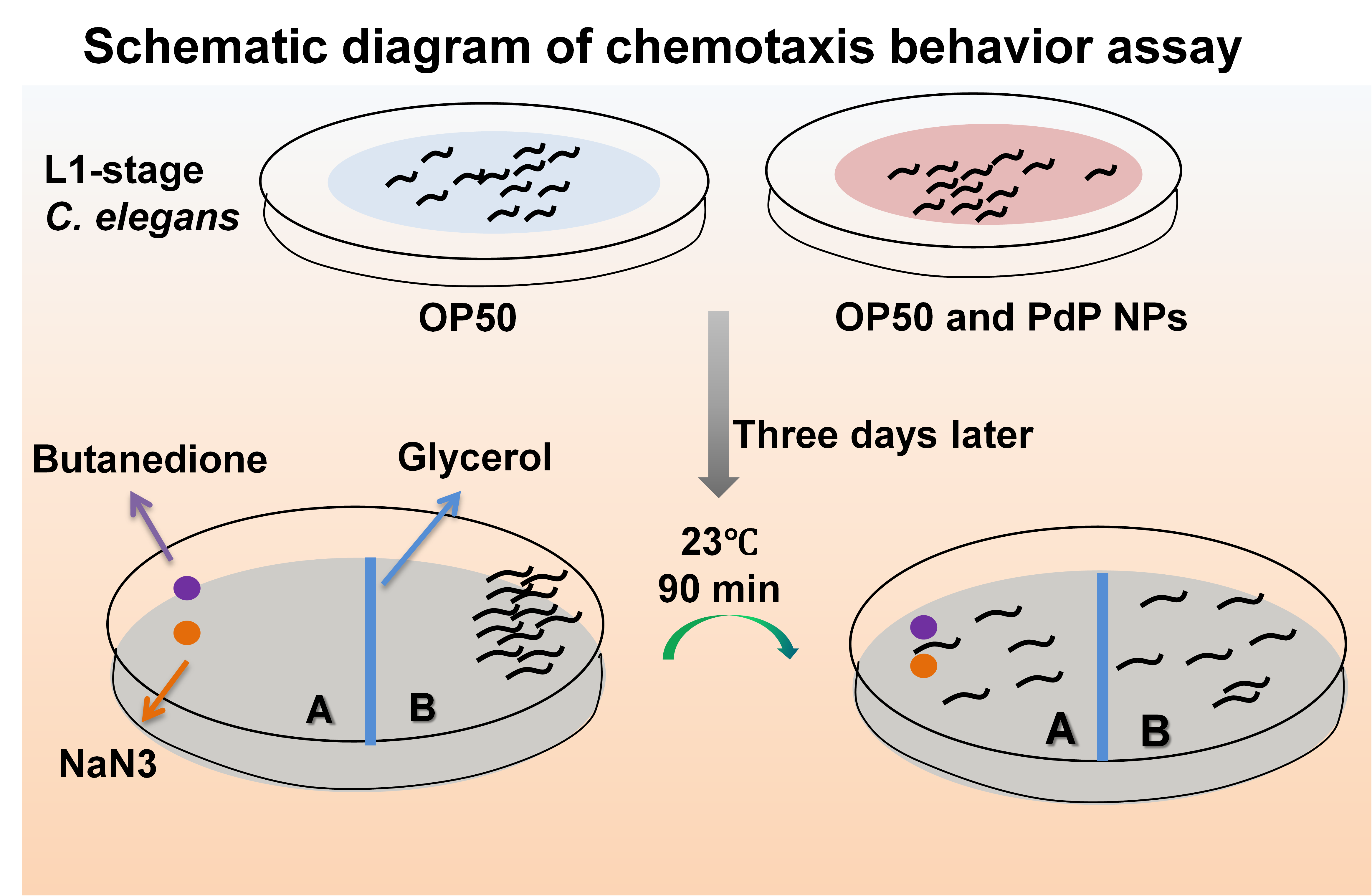


**Figure S24.** Schematic diagram for chemotaxis behavior assay system. The nematodes located at the B region could be attracted towards the A region by butanedione. Once the nematodes approached the A region, they will be paralyzed by NaN_3_. The chemosensory index (B/A+B) were calculated by counting the number of worms in areas A and B separately after 90 minutes of test.


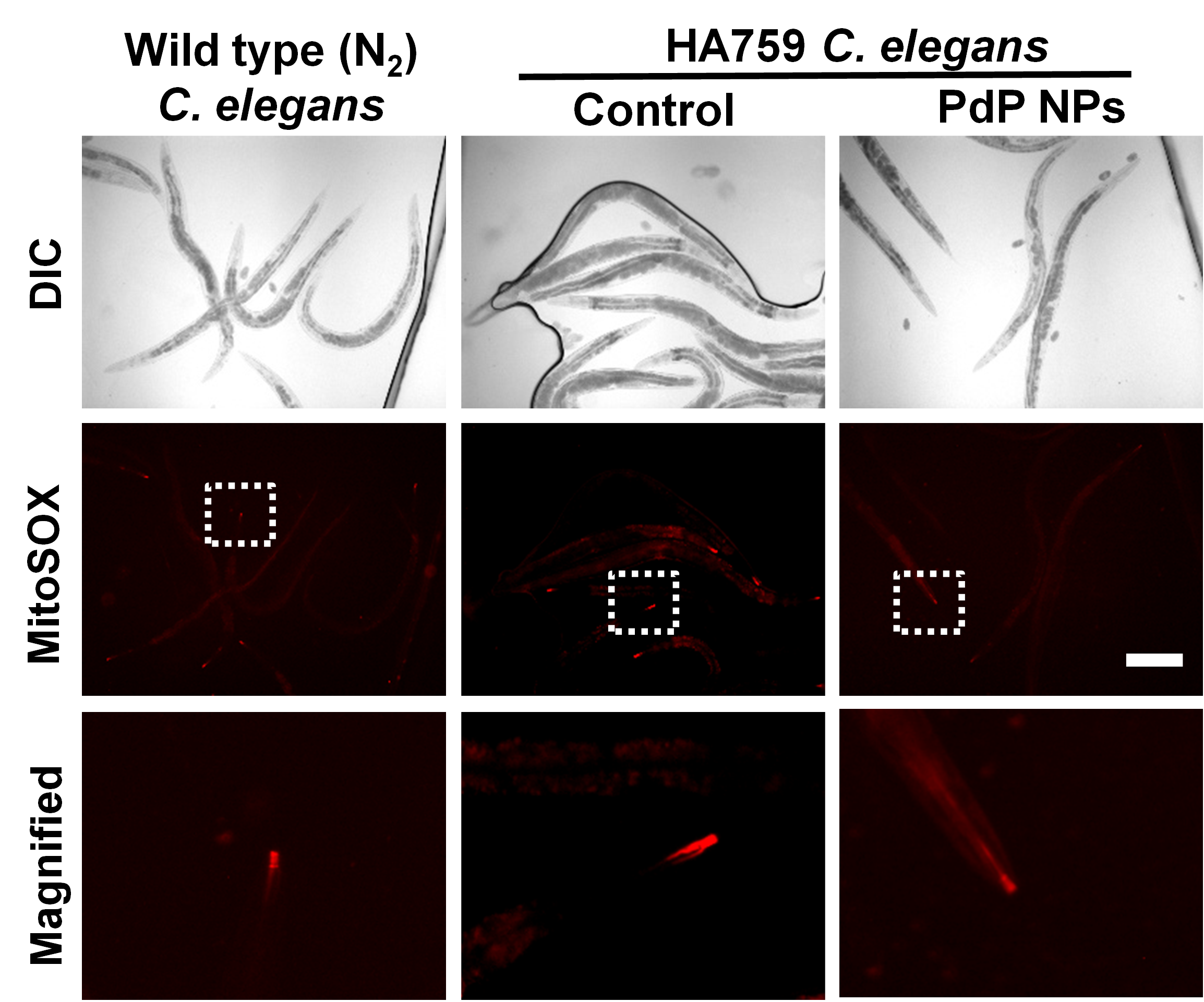


**Figure S25.** Relatively quantified levels of mitochondrial ROS on nematode head with MitoSOX probe using fluorescent microscopy in wild type (N_2_) and HA759 *C. elegans*. Scale bar = 200 μm.

**Table S1.** **Primers Used in RT-qPCR**

| **Gene name** | **Sequence (5’-3’)** |
| --- | --- |
| *cts-1*-rv | GGTACAGGTTGCGATAGATGATAGC |
| *cts-1*-fw | CTCGACAACTTCCCAGATAACC |
| *cox-4*-rv | AGGTTGGCGGCAGTTCTGGG |
| *cox-4*-fw | GCCCCAATTCGCGCCAAGGA |
| *hxk-1*-rv | CTAGAGATGACGTCACACACTTCTC |
| *hxk-1*-fw | GTGCGACGAGTACTTTCTCAACTG |
| *pyc-1*-rv | GTGATCATACATCCTGGTCTACTGC |
| *pyc-1*-fw | TCCAACTACTCCTCTTGCTACTGAC |
| *fzo-1*-rv | AGTCGGCATTCCCCTGATTCCG |
| *fzo-1*-fw | TCTGCAGGTTGAAGGTTCAGAAGGC |
| *opa-1*-rv | CTTGGCCATCCATTCTGCCCA |
| *opa-1*-fw | TCGCGGCTAGAACGTGGTATGA |
| *ddp-1-*rv | AGTGCTCGACCATGAAGTTG |
| *ddp-1*-fw | AACAAGTGCACACGCTCA |
| *suclg-1*- rv | CAGCTGATCCTCCGATTTCT |
| *suclg-1*- fw | GTCGGATTCGGACAGACTTT |
| *mrpl-47-*rv | ACTCGTGGAGCTCCTCTCTTGA |
| *mrpl-47-*fw | CGACGACGATGCCTACGTGA |
| *mrps-14-*rv | TTGGACTCCACTGAGAGCTG |
| *mrps-14-*fw | CGATCATCCTCGTCTGATTC |
| *mfn-1-* rv | TGCAGGAACCTGGAATATGA |
| *mfn-1-*fw | GATCCAGCAAATAGGCGAAT |
| *F17E5.2-rv* | CCTTCATGAAATTCGGTGTG |
| *F17E5.2-* fw | TCGAACAAGGCTTCAAGCTA |
| *F58F12.1-*rv | TGATCAATGCTTCAGCAACTT |
| *F58F12.1-*fw | CTTGATGCTGCCCAAAGAG |
| D2030.4*-rv* | TTCAAGCAGTGGCTTCTTTG |
| D2030.4*-*fw | TGGAGCTTGGGATAAATGTG |
| *tag-174-rv* | TAGTGCTTCTCACGGTCAGC |
| *tag-174-*fw | TCGAGTACGCCTTCTTGAAC |
| *cyc-2.1-rv* | TTCTTGAGTCCAGCGAACAC |
| *cyc-2.1-*fw | CGCTGCTAACAAGAACAAGG |
| *act-1*-rv | GTAGCAGAGCTTCTCCTTGATGTC |
| *act-1*-fw | GCTGGACGTGATCTTACTGATTACC |
| *act-3*-rv | GGTGGTTCTCCGGAAAGAA |
| *act-3*-fw | TGCGACATTGATATCCGTAAGG |
| *nd-1*-rv | AAGCTTGTGCTAATCCCATAAATGT |
| *nd-1*-fw | AGCGTCATTTATTGGGAAGAAGAC |
| *mtce.26*-rv | CAGGGTGCCCCATTGTTCTT |
| *mtce.26*-fw | GGTTGTGGGACTAGGTGAACA |
